# Comparison of Selected Pro-Health Biologically Active Chemical Compounds in *Salviae herba* from Selected Species

**DOI:** 10.3390/molecules31091425

**Published:** 2026-04-26

**Authors:** Mirosława Chwil, Jarmila Neugebauerová, Renata Matraszek-Gawron, Tadeusz Paszko

**Affiliations:** 1 Department of Botany and Plant Physiology, University of Life Sciences in Lublin, Akademicka 15 Street, 20-950 Lublin, Poland; miroslawa.chwil@up.lublin.pl; 2 Department of Vegetable Growing and Floriculture, Faculty of Horticulture, Mendel University in Brno, Valtická 337 Street, 691 44 Lednice, Czech Republic; jarmila.neugebauerova@mendelu.cz; 3 Department of Chemistry, University of Life Sciences in Lublin, Akademicka 15 Street, 20-950 Lublin, Poland; tadeusz.paszko@up.lublin.pl

**Keywords:** *Salvia officinalis*, *Salvia officinalis* subsp. *lavandulifolia*, *Salvia sclarea*, phytotherapy, phytochemicals, amino acids, fatty acids, flavonoids, phenolic compounds, antioxidant activity

## Abstract

Pharmaceutical, cosmetic, and food industries have contributed to the increasing interest in herbal phytochemicals. *Salvia*, a multifunctional culinary herb, meets phytotherapeutic requirements in the treatment of heartburn, excessive sweating, flatulence, and mouth, throat, and skin inflammatory conditions. *Salviae folium* is used in conductive education, i.e., a unique rehabilitation method for individuals with neurological and motor disorders designed to help in learning to perform activities independently. The comparative analysis of bioactive chemical compounds in *S. officinalis*, *S. officinalis* subsp. *lavandulifolia*, and *S. sclarea* herb showed that *S. officinalis* had the highest concentration of exogenous amino acids (53 mg·g^−1^ DW), with a predominance of lecithin and phenylalanine, and endogenous amino acids were dominated by aspartic and glutamic acids. *S. officinalis* subsp. *lavandulifolia* was the richest source of omega-3, omega-6, and omega-9 fatty acids, followed by *S. officinalis* and *S. sclarea*. The vitamin C content was 4.9 (*S. sclarea*)–14.4 mg·100 g^−1^ DW (*S. officinalis*). Phenolic acids were dominated by rosmarinic acid (*S. officinalis* > *S. officinalis* sub. *lavandulifolia* > *S. sclarea*) and ferulic acid (*S. officinalis* > *S. sclarea* > *S. officinalis* sub. *lavandulifolia*). *Salvia sclarea* is a rich source of *p*-coumaric acid. Among non-phenolic organic acids, the highest content of quinic and malic acids was found in *S. sclarea* and *S. officinalis*, respectively. The level of *o*-dihydroxyphenols was 2140 (*S. officinalis*)-2222 mg CAE·100·g^−1^ DW (*S. sclarea)*. The flavonoid content was 610 (*S. officinalis* subsp. *lavandulifolia*)-347 mg RU·100 g^−1^ DW (*S. sclarea*). Flavonoids, flavonols, and flavanones were dominated by apigenin, kaempferol, and hesperidin, respectively. These metabolites may be potential components in phytotherapeutic products.

## 1. Introduction

Global herbal medicine is currently developing due to the growing consumer demand for products containing health-enhancing phytochemicals [1,2,3,4,5,6,7]. Various herbal products are available on the market: pharmaceuticals, nutraceuticals, functional foods, dietary supplements, cosmetic ingredients, and medications [2,3,8]. The popularity of herbs is growing due to their effectiveness, non-toxic nature, and minimal side effects [8].

### 1.1. Development of Herbal Medicine

The public awareness of the activity and therapeutic properties of phytochemicals as well as their application as part of innovative methods used in modern medical healthcare practices is increasing [7]. As a result, the popularity of herbal products and medications as well as public awareness are on the increase in many countries in Europe, mainly in Poland [9,10,11,12], Romania [13], Great Britain [14,15], Belarus [16], Ukraine Mattalia et al. 2022 [13], Lithuania [16,17,18,19,20], and Northern Cyprus [21]. Increasing interest has also been reported in Asia, e.g., in India [22,23,24], Pakistan [25], Saudi Arabia [6,26,27], Turkey [İDUĞ 2023 [28], in Africa (Nigeria) [29], Australia [30], and North America (Canada and the United States) [31,32].

The needs of modern medicine as well as pharmaceutical and cosmetic industries have contributed to a significant increase in the interest in traditional medicine and the use of active chemical compounds derived from various species of herbs, including the genus *Salvia*. Many *Salvia* species are investigated in experimental studies in various biological and clinical models to identify their pharmacological effects in the phytotherapy of many diseases [22,33,34].

### 1.2. Application of Salvia officinalis, S. officinalis subsp. lavandulifolia, and Salvia sclarea in Phytotherapy

*Salvia officinalis* L., *S. officinalis* subsp. *lavandulifolia*, and *Salvia sclarea* L. have long been used as valuable medicinal plants in traditional medicine in many countries [35]. Various raw materials, i.e., *Salviae folium*, *Salviae herba*, and *Salviae rhizoma* derived from e.g., *S. officinalis* [11,36,37,38,39,40,41,42], *S. officinalis* subsp. *lavandulifolia* [43,44,45], and *S. sclarea* [46,47,48,49,50,51,52,53,54,55] are commonly used in phytotherapy.

#### 1.2.1. *Salvia officinalis* L.

Described by Hippocrates, *S. officinalis* was used in ancient Greece [56]. At present, the species is used as part of phytotherapy in many diseases. A mouthwash with *S. officinalis* extract was documented to alleviate aphthous symptoms, mitigate pain, and reduce the size of ulcers in gingivitis [57]. It also reduced oral discomfort in cancer patients undergoing palliative oncological treatment [40]. Lozenges containing 1893 mg of *S. officinalis* extract and 4000 mg of *Echinacea purpurea* extract used once daily for 4 days relieved sore throat symptoms [58].

Flavonoids, sterols, volatile oils, saponins, and carbohydrates derived from *S. officinalis* herb inhibited the growth of *Helicobacter pylori*. The most potent antibacterial and anti-inflammatory effects were exerted by ethanol extracts at a concentration of 3.9 μg·ml^−1^ and essential oils applied at 15.6 μg·ml^−1^ (in vitro, in silico). The metabolites were dominated by (%) eucalyptol (50.1), carnosic acid (37.7), epirosmanol (20.7), camphor (17.8), 12-*O*-methylcarnosol (6.2), and carnosol (3.3) [42]. Anti-inflammatory, antioxidant, and chemopreventive properties were exhibited by manool isolated from *S. officinalis*; it regulated signalling pathways and prevented colon carcinogenesis [59]. Additionally, an 8-week treatment with *S. officinalis* extract at a dose of 330 mg/day reduced the body mass index (BMI) and blood pressure and improved markers of insulin resistance in patients with euglycemia and PCOS [60].

Rosmarinic acid from *S. officinalis* has been documented to exert neuroprotective and antioxidant effects and prevent the formation of β-amyloid plaques, which are key agents in the pathogenesis of Alzheimer’s disease [56]. Hydroalcoholic extracts and essential oils obtained from leaves of this species had a positive effect on the memory of patients with this disease [34]. Also in Alzheimer’s disease, the active chemical compounds from *S. officinalis* in combination with curcumin, a low-fat diet, NuAD-Trail, and soy lecithin proved beneficial; they were more effective than the allopathic treatment alone [61,62].

#### 1.2.2. *Salvia officinalis* subsp. *lavandulifolia* (Vahl) Gams

*S. officinalis* subsp. *lavandulifolia* is an important medicinal plant with a long tradition of use in folk medicine for treatment of many diseases [63]. The medicinal use of this plant is based on its content (%) of 1,8-cineole (24.3–34), camphor (23.5–28.8), camphene (4.9–6.4), and α-pinene (4.3–6.6) [45]. In some countries, e.g., Spain, sage is cultivated on a large scale for extraction of its essential oil and manufacture of other products to be used in pharmaceutical, cosmetic, and food industries. *S. officinalis* subsp. *lavandulifolia* is used in phytotherapy as infusions, chewing preparations, and mouthwashes [44,63]. It has analgesic, antitussive, antimigraine, antispasmodic, sedative, and antioxidant properties [43,63]. Additionally, it exhibits antibacterial activity against *Staphylococcus aureus*. The minimal inhibitory concentration (MIC) of linalool, camphor, α-pinene, linalyl acetate, and essential oil against this skin pathogen is in the range of 2.5–10 mg·ml^−1^. Biologically active compounds and essential oil from this species have high antioxidant activity exceeding 30 mg·ml^−1^ (determined with the use of 1,1-diphenyl-2-picrylhydrazyl (DPPH)), which indicates antiseptic activity (in vitro) [43]. The antifungal activity of these compounds against *Botrytis cinerea* and *Candida albicans* has also been evidenced [44,45].

#### 1.2.3. *Salvia sclarea* L.

*Salvia sclarea* L. is used in folk medicine for treatment of various diseases, e.g., gingivitis and aphthous stomatitis. In periodontitis, ethanol *S. sclarea* extracts have been shown to reduce the levels of IL-1β, IL-6, TNF-α, and the number of inflammatory cells, increase the number of fibroblasts, and alleviate inflammation symptoms. The dominant rosmarinic acid in the extract has antioxidant and anti-inflammatory activity; therefore, it may be a potential therapeutic agent in periodontal diseases [64].

Diterpenoids, i.e., aethiopinone and salvipisone, isolated from *S. sclarea* hairy roots exhibited high cytotoxic activity against HL-60 and NALM-6 leukaemia cells (in vitro). These compounds induced apoptosis via the caspase-3 pathway in a concentration-dependent manner. The high efficacy of the *S. sclarea* diterpenoids against drug-resistant leukaemia cells suggests that they may be used for treatment in drug-resistant patients [47].

Linalyl (50.4%) and linalyl acetate (50.4%) dominate in the essential oil of *S. sclarea* cultivated in Iran. Linalyl acetate can be used as part of complementary phytotherapy for diabetes [52]. Essential oil from *S. sclarea* grown in Ukraine was effective against common bacterial pathogens: *Enterococcus faecalis*, *Escherichia coli*, *Staphylococcus aureus*, and *Streptococcus pyogenes* and against *Candida albicans* yeast. The high levels of linalool (39%) and linalyl acetate (45.5%) were shown to be involved in its antimicrobial properties. *S. sclarea* can be used in the pharmaceutical industry and for preservation of food and cosmetic products [65].

Many investigations have been conducted in various biological models in the field of phytotherapy and the application of the species in the pharmaceutical, cosmetic, and food industries and as functional food, dietary supplements, nutraceuticals, herbal medications, and other food and cosmetic products. However, the literature does not provide a comparative analysis of the chemical profile (especially the profile of bioactive compounds with pro-health activity in humans) of the herb of *S. officinalis*, *S. officinalis* sub. *lavandulifolia*, and *S. sclarea* grown in the South Moravian region of the Czech Republic (Lednice). Therefore, an attempt was made to supplement this information.

The aim of the study was to determine the content of selected metabolites: (i) total protein, (ii) total fat, (iii) ash, (iv) vitamin C, (v) total flavonoids, (vi) phenolic acids, (vii) malic acid, (viii) quinic acid, and (ix) *orto*-dihydroxyphenols. Additionally, qualitative and quantitative analysis was performed to detect (x) fatty acids, (xi) amino acids, (xii) phenolic acids, and (xii) flavonoids in the herb of *Salvia officinalis* L., *Salvia officinalis* sub. *lavandulifolia* (Vahl) Gams, and *Salvia sclarea* L. A comparative analysis of these parameters was carried out and dominant bioactive compounds with pro-health effects on the human organism were identified.

## 2. Results

**Figure 1:**
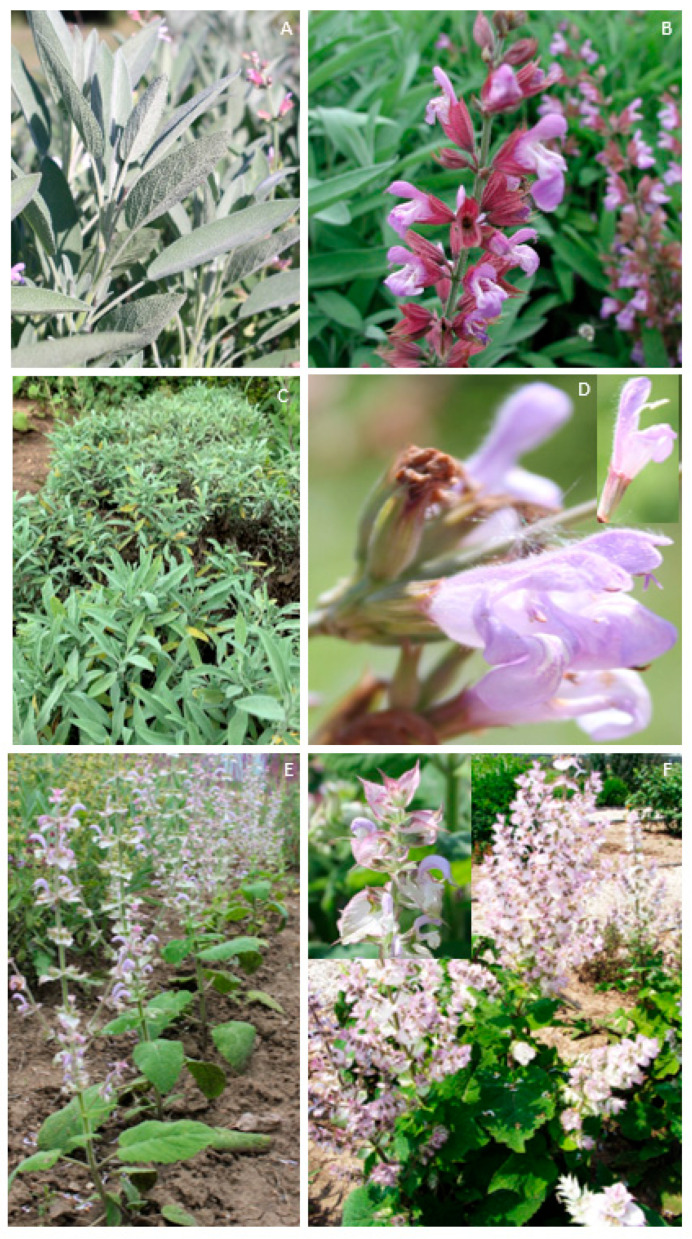
(**A**–**F**). *S. officinalis* (**A**,**B**), *S. officinalis* subsp. *lavandulifolia* (**C**,**D**), and *S. sclarea* (**E**,**F**), (**A**,**C**,**E**)—aboveground parts, (**B**)—inflorescence, (**D**)—flowers, (**F**)—plants in full bloom.

A comparative analysis of selected bioactive chemical compounds was carried out in *Salvia herba* raw material from *S. officinalis*, *S. officinalis* subsp. *lavandulifolia*, and *Salvia sclarea* in the early flowering phase (Figure 1A–F).

### 2.1. Protein, Fat, Ash Content, and Moisture in Salviae Herba Samples

**Figure 2:**
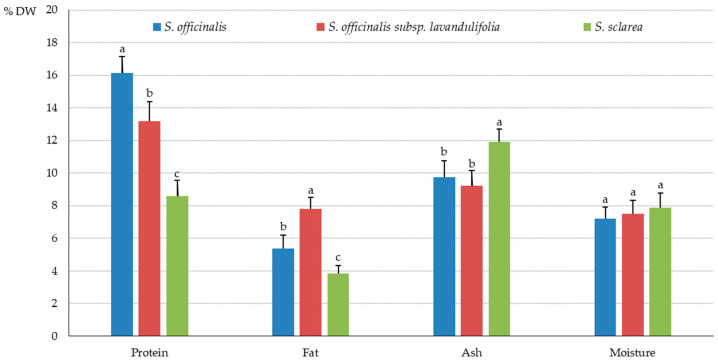
Percent content of protein, fat, ash, and moisture in the dry matter of *Salviae herba* of *S. officinalis*, *S. officinalis* subsp. *lavandulifolia*, and *S. sclarea*. Notes: Means of each parameter followed by the same letter do not differ at a significance level of α = 0.05. Vertical bars represent the standard deviations of the means.

The highest value of total protein in the herb of the analysed *Salvia* species was recorded in *S. officinalis* (16.14 ± %), followed by *S. officinalis* subsp. *lavandulifolia* (13.17%) and *S. sclarea* (8.57%). The highest total fat content was found in the herb of *S. officinalis* subsp. *lavandulifolia* (7.80%), a moderate level was detected in *S. officinalis* (5.39%), and the lowest amount was found in *S. sclarea* (3.84%). The ash content was similar in the herb of *S. officinalis* subsp. *lavandulifolia* and *S. officinalis* (9.24 and 9.76%, respectively), but it was crucially lower than in *S. sclarea* (11.49%). The moisture content in the herb samples varied slightly between the species from 7.22% (*S. officinalis*) to 7.89% (*S. sclarea*) (Figure 2).

### 2.2. Protein Amino Acids

**Figure 3:**
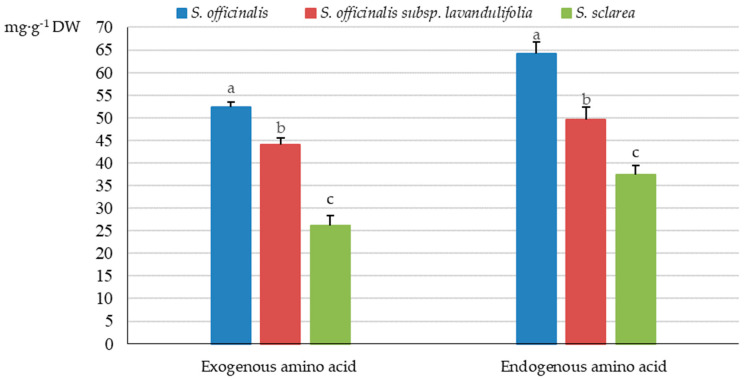
Content of exogenous and endogenous protein amino acids in the *Salviae herba* of *S. officinalis*, *S. officinalis* subsp. *lavandulifolia*, and *S. sclarea*. Notes: Means for exo- and endogenous amino acids followed by the same letter do not differ at a significance level of α = 0.05. Vertical bars represent the standard deviations of the means.

In the analysed taxa, the highest total protein amino acid content was determined in the herb of *S. officinalis* (116.50 mg·g^−1^ DW); it was approximately 20% and 46% lower in *S. officinalis* subsp. *lavandulifolia* (93.5 mg·g^−1^ DW) and *S. sclarea* (63.4 mg·g^−1^ DW), respectively (Figure 3).

#### 2.2.1. Exogenous Amino Acids

**Figure 4:**
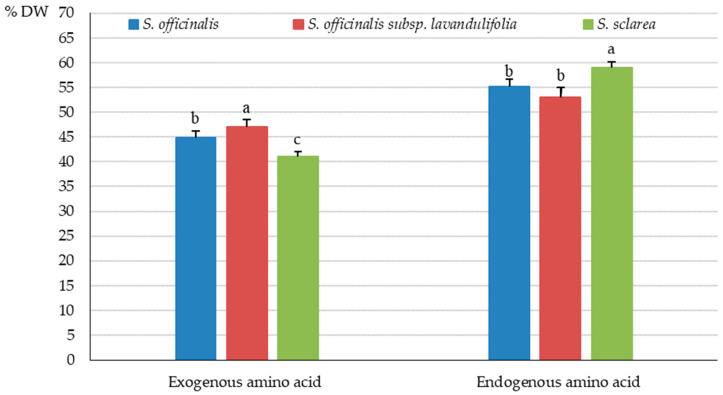
Percentage share of the content of exogenous and endogenous amino acids in the total pool of protein amino acids in the *Salviae herba* of *S. officinalis*, *S. officinalis* subsp. *lavandulifolia*, and *S. sclarea*. Notes: Means for exo- and endogenous amino acids followed by the same letter do not differ at a significance level of α = 0.05. Vertical bars represent the standard deviations of the means.

The analysis of the protein amino acid group in the *Salvia* taxa showed the highest content of exogenous amino acids in *S. officinalis* (52.27 mg·g^−1^ DW), moderate amounts in *S. officinalis* subsp. *lavandulifolia* (43.97 mg·g^−1^ DW), and the lowest levels in *S. sclarea* (26.05 mg·g^−1^ DW) (Figure 3), which accounted for 44.79, 47.03, and 41.09% of the total amino acid pool, respectively (Figure 4). The proportion of endogenous amino acids in the total pool present in the herb of the studied taxa exceeded the percent content of exogenous amino acids and was 58.91% in *S. sclarea*, 55.13% in *S. officinalis*, and 52.97% in *S. officinalis* subsp. *lavandulifolia* (Figure 4).

**Figure 5:**
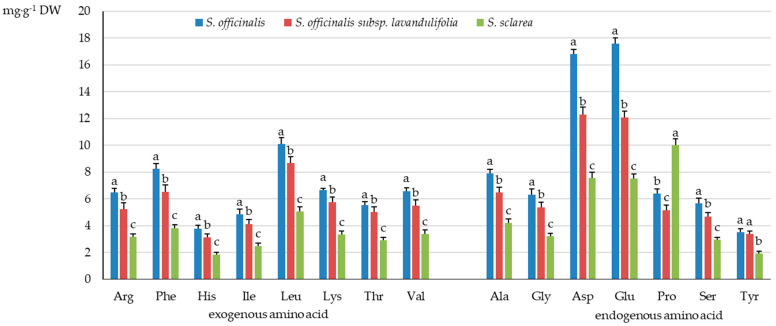
Content of each exogenous and endogenous amino acid in the *Salviae herba* of *S. officinalis*, *S. officinalis* subsp. *lavandulifolia*, and *S. sclarea*. Notes: Means of each amino acid followed by the same letter do not differ at a significance level of α = 0.05. Vertical bars represent the standard deviations of the means.

Similar groups of exogenous protein amino acids dominated in the herb of *S. officinalis* subsp. *lavandulifolia*, *S. officinalis*, and *S. sclarea*. The content of each exogenous amino acid differed considerably between the analysed samples of all three *Salviae herba* raw materials and was the lowest in *S. sclarea*, moderate in *S. officinalis* subsp. *lavandulifolia*, and the highest in *S. officinalis*. The highest concentration among the exogenous amino acids in the three analysed taxa was determined for the aliphatic amino acid leucine (Leu), which ranged from 5.06 to 10.10 mg·g^−1^ DW. The second most abundant amino acid was aromatic amono-acid phenylalanine (Phe) with the content ranging from approximately 3.84 to 8.25 mg·g^−1^ DW, followed by valine (Val) from the same group as leucine with a concentration between 3.39 and 6.59 mg·g^−1^ DW (Figure 5).

**Figure 6:**
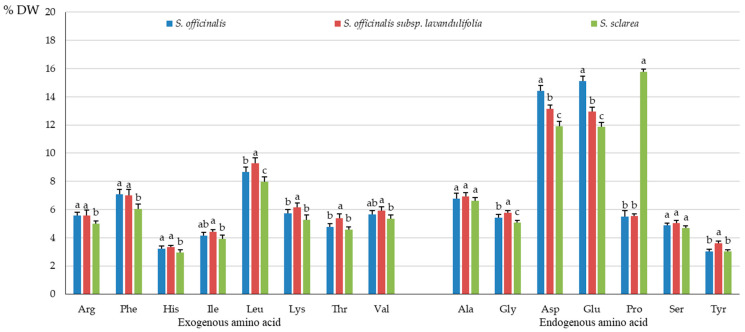
Percentage share of the content of each exogenous and endogenous amino acid in the total pool of protein amino acids in the *Salvia herba* of *S. officinalis*, *S. officinalis* subsp. *lavandulifolia*, and *S. sclarea*. Notes: Means of each amino acid followed by the same letter do not differ at a significance level of α = 0.05. Vertical bars represent the standard deviations of the means.

The percentage share of the content of these three dominant exogenous amino acids, i.e., Leu, Phe, and Val, in the total pool of protein amino acids in the *Salvia herba* accounted for 7.98 (*S. sclarea*)–9.27% (*S. officinalis* subsp. *lavandulifolia*), 6.06 (*S. sclarea*)–7.08% (*S. officinalis*), and 5.35 (*S. sclarea*)–5.9% (*S. officinalis* subsp. *lavandulifolia*), respectively (Figure 6).

The two exogenous amino acids with the lowest level recorded in the *Salviae herba* were isoleucine (Ile) (2.49–4.85 mg·g^−1^ DW) and histidine (His) (1.86–3.77 mg·g^−1^ DW) (Figure 5) with the percentage share of its content in the range of 3.93–4.42% and 2.93–3.34%, respectively (Figure 6). The percentage share of Ile and His in *S. officinalis* subsp. *lavandulifolia* and *S. officinalis* was comparable but remarkably exceeded the values recorded in *S. sclarea*.

#### 2.2.2. Endogenous Protein Amino Acids

As in the case of the exogenous amino acids, the *S. officinalis* subsp. *lavandulifolia* herb was characterised by markedly higher content of endogenous amino acids than in *S. sclarea* and a notably lower level than in *S. officinalis*. Only the proline (Pro) level in *S. officinalis* (6.40 mg·g^−1^ DW) exceeded its content in *S. officinalis* subsp. *lavandulifolia* (5.17 mg·g^−1^ DW), but was lower than in *S. sclarea* (10.00 mg·g^−1^ DW). The tyrosine (Tyr) contents in the herb of *S. officinalis* subsp. *lavandulifolia* and *S. officinalis* were similar (3.38 and 3.52 mg·g^−1^ DW) but higher than in the *S. sclarea* herb (1.91 mg·g^−1^ DW) (Figure 5).

In the group of endogenous amino acids, the highest values ranging from 7.52 to 17.60 mg·g^−1^ DW were determined for glutamic acid (Glu). A slightly lower concentration was exhibited by aspartic acid (Asp) ranging from 7.55 to 16.80 mg·g^−1^ DW, followed by alanine (Ala) with the level ranging from 4.20 to 7.90 mg·g^−1^ DW (Figure 5). These dominant endogenous amino acids Glu and Asp accounted for 11.86 (*S. sclarea*)–15.11% (*S. officinalis*) and 11.91 (*S. sclarea*)–14.42% (*S. officinalis*) of the total pool of protein amino acids, respectively, and proline constituted 5.5–15.8% (Figure 6). The two endogenous amino acids with the lowest level recorded in the *Salviae herba* were serine (Ser) (2.96–5.67 mg·g^−1^ DW) and tyrosine (Tyr) (1.91–3.52 mg·g^−1^ DW) (Figure 5), with the percentage value range of 4.67 (*S. sclarea*)–5.03% (*S. officinalis* subsp. *lavandulifolia*) and 3.01 (*S. sclarea*)–3.62% (*S. officinalis* subsp. *lavandulifolia*), respectively (Figure 6).

The nomenclature, abbreviations, symbols, and linear and structural formulas of fifteen amino acids identified in the *Salviae herba* of the three analysed taxa are presented in Table 1.

### 2.3. Fatty Acid Profile

#### 2.3.1. Saturated Fatty Acids (SFAs), Monounsaturated Fatty Acids (MUFAs), and Polyunsaturated Fatty Acids (PUFAs)

**Figure 7:**
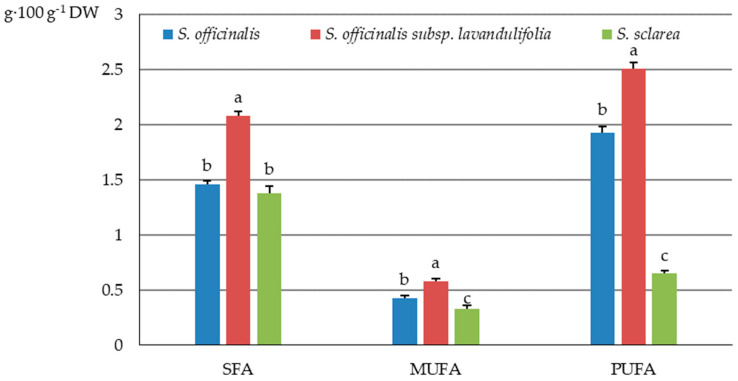
Content of saturated fatty acids (SFAs), mono unsaturated fatty acids (MUFAs), and polyunsaturated fatty acids (PUFAs) in the *Salviae herba* of *S. officinalis*, *S. officinalis* subsp. *lavandulifolia*, and *S. sclarea*. Notes: For each fatty acid group, means followed by the same letter do not differ at a significance level of α = 0.05. Vertical bars represent the standard deviations of the means.

The highest content of SFAs, MUFAs, and PUFAs was determined in the *S. officinalis* subsp. *lavandulifolia* herb (2.08, 0.58, and 2.51 g·100 g^−1^ DW, respectively), followed by *S. officinalis* (1.46, 0.43, and 1.93 g·100 g^−1^ DW) and *S. sclarea* (1.38, 0.33, and 0.65 g·100 g^−1^ DW) (Figure 7).

**Figure 8:**
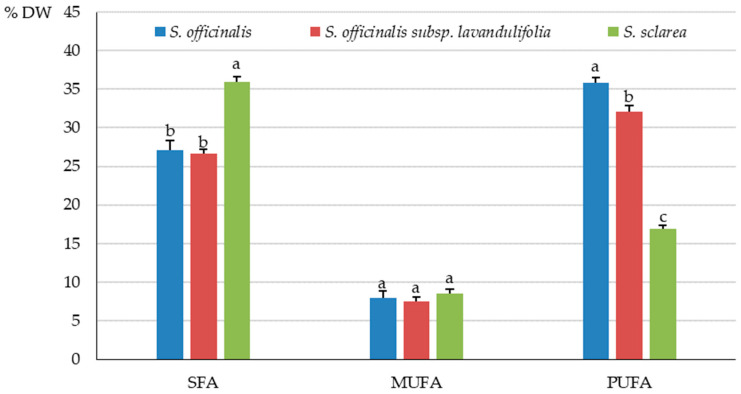
Percentage share of saturated fatty acids (SFAs), mono unsaturated fatty acids (MUFAs), and polyunsaturated fatty acids (PUFAs) in the total pool of fat extracted from *Salviae herba* of each taxa of *S. officinalis*, *S. officinalis* subsp. *lavandulifolia*, and *S. sclarea*. Notes: For each fatty acid group, means followed by the same letter do not differ at a significance level of α = 0.05. Vertical bars represent the standard deviations of the means.

The fatty acids identified in the *S. sclarea* herb were represented by 36% of SFAs in the total pool of fat extracted from samples of these taxa; they considerably exceeded the level in *S. officinalis* (27.2%) and *S. officinalis* subsp. *lavandulifolia* (26.7%). The percentage share of MUFAs in the total pool of fat did not differ markedly, and their value ranged from 7.5% (*S. officinalis* subsp. *lavandulifolia*) to 8.5% (*S. sclarea*). The percentage share of PUFAs in the herb of *S. officinalis* subsp. *lavandulifolia* (32%) notably exceeded those recorded in *S. sclarea* (17%), but was lower than in *S. officinalis* (36%) (Figure 8).

#### 2.3.2. Omega Fatty Acid Family

##### Omega-3 Fatty Acids

**Figure 9:**
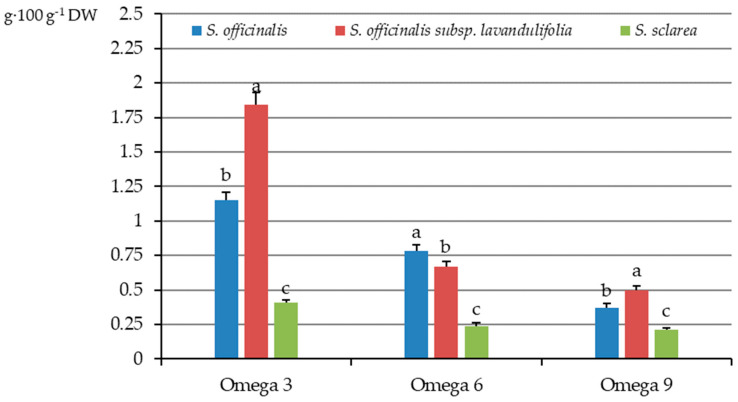
Content of omega 3, omega 6, and omega 9 acids in *Salviae herba.* Notes: In each fatty acid group, means followed by the same letter do not differ at a significance level of α = 0.05. Vertical bars represent the standard deviations of the means.

Among the three main groups of fatty acids (omega-3, omega-6, and omega-9) omega-3 acids, with the first double bond located at the third carbon from the methyl end of the carbon chain, were the most abundant in the analysed samples of the three *Salviae herba* types. The highest content of this fatty acid group was found in the herb of *S. officinalis* subsp. *lavandulifolia* (1.84 g·100 g^−1^ DW), a moderate level was determined in *S. officinalis* (1.15 g·100 g^−1^ DW), and the lowest amount was found in *S. sclarea* (0.41 g·100 g^−1^ DW), with a significant drop between the taxa by approximately 38 and 78%, respectively (Figure 9).

**Figure 10:**
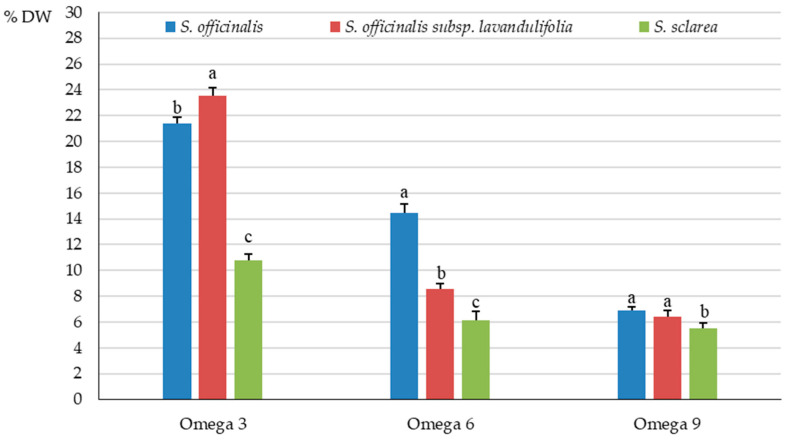
Percentage share of omega 3, omega 6, and omega 9 acids in the pool of fat extracted from each *Salviae herba* taxon. Notes: In each fatty acid group, means followed by the same letter do not differ at a significance level of α = 0.05. Vertical bars represent the standard deviations of the means.

A similar tendency was shown for the percentage share of omega 3 acids in the total pool of fats. The value of this parameter in *S. officinalis* (21.4%) considerably exceeded that in *S. sclarea* (10.8%) but was distinctly lower than in *S. officinalis* subsp. *lavandulifolia* (23.6%) (Figure 10).

**Figure 11:**
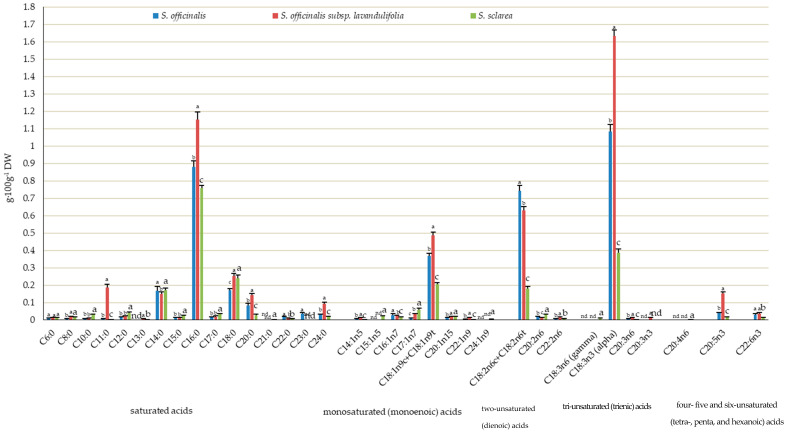
Content of fatty acids in *Salviae herba.* Notes: C6:0 (caproic acid), C8:0 (caprylic acid), C10:0 (capric acid), C11:0 (undecanoic acid), C12:0 (lauric acid), C13:0 (tridecanoic acid), C14:0 (myristic acid), C15:0 (pentadecylic acid), C16:0 (palmitic acid), C17:0 (margaric acid), C18:0 (stearic acid), C20:0 (arachidic acid), C21:0 (heneicosylic acid), C22:0 (behenic acid), C23:0 (tricosylic acid), C24:0 (lignoceric acid), C14:1n5 (myristoleic acid), C15:1n5 (10(Z)-pentadecenoic acid), C16:1n7 (palmitoleic acid), C17:1n7 ((10Z)-10-heptadecenoic acid), C18:1n9c (oleic acid), C18:1n9t (elaidic acid), C20:1n15 ((5Z)-eicosenoic acid), C22:1n9 (eruic acid), C24:1n9 (nervonic acid), C18:2n6c (linoleic acid), C18:2n6t (linoelaidic acid), C20:2n6 (n-6 eicosadienoic acid), C22:2n6 (13,16-docosadienoate acid), C18:3n6 gamma (GLA, γ-linolenic acid), C18:3n3 alpha (ALA, α-linolenic acid), C20:3n6 (DGLA, dihomo-γ-linolenic acid), C20:3n3 (*Z*-11,14,17-eicosatrienoic acid), C20:4n6 (arachidonic acid), C20:5n3 (EPA, timnodonic acid), C22:6n-3 (DHA, cervonic acid). Means of each fatty acid followed by the same letter do not differ at a significance level of α = 0.05. nd—not determined.

**Figure 12:**
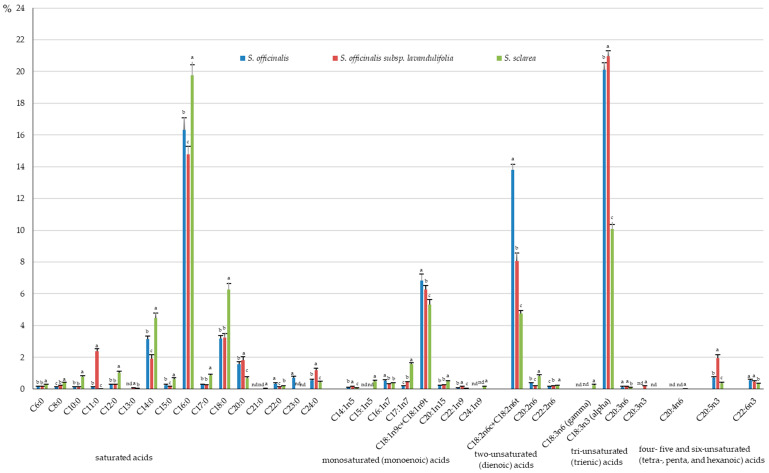
Percentage share of fatty acids in the total pool of fat extracted from the *Salviae herba* of the studied taxa. Notes: C6:0 (caproic acid), C8:0 (caprylic acid), C10:0 (capric acid), C11:0 (undecanoic acid), C12:0 (lauric acid), C13:0 (tridecanoic acid), C14:0 (myristic acid), C15:0 (pentadecylic acid), C16:0 (palmitic acid), C17:0 (margaric acid), C18:0 (stearic acid), C20:0 (arachidic acid), C21:0 (heneicosylic acid), C22:0 (behenic acid), C23:0 (tricosylic acid), C24:0 (lignoceric acid), C14:1n5 (myristoleic acid), C15:1n5 (10(Z)-pentadecenoic acid), C16:1n7 (palmitoleic acid), C17:1n7 ((10Z)-10-heptadecenoic acid), C18:1n9c (oleic acid), C18:1n9t (elaidic acid), C20:1n15 ((5Z)-eicosenoic acid), C22:1n9 (eruic acid), C24:1n9 (nervonic acid), C18:2n6c (linoleic acid), C18:2n6t (linoelaidic acid), C20:2n6 (n-6 eicosadienoic acid), C22:2n6 (13,16-docosadienoate acid), C18:3n6 gamma (GLA, γ-linolenic acid), C18:3n3 alpha (ALA, α-linolenic acid), C20:3n6 (DGLA, Dihomo-γ-linolenic acid), C20:3n3 (*Z*-11,14,17-eicosatrienoic acid), C20:4n6 (arachidonic acid), C20:5n3 (EPA, timnodonic acid), C22:6n-3 (DHA, cervonic acid). Means of each fatty acid followed by the same letter do not differ at a significance level of α = 0.05. nd—not determined.

The qualitative analysis of the composition of omega-3 polyunsaturated fatty acids (PUFAs) allowed separation and identification of the following compounds in the herb of the three taxa: α-linolenic acid (C18:3n3 alpha), EPA timnodonic acid (C20:5n3), and DHA cervonic acid (22:6n3). The content of each of these acids differed markedly between the taxa, being the lowest in *S. sclarea* and the highest in *S. officinalis* subsp. *lavandulifolia* with the range of 0.037–1.634, 0.014–0.153, and 0.013–0.037 g·100 g^−1^ DW, respectively. A representative of the omega-3 group, i.e., *Z*-11,14,17-eicosatrienoic acid C20:3n3 (0.013 g·100 g^−1^ DW), was identified only in the herb of *S. officinalis* subsp. *lavandulifolia* (Figure 11), with the percentage share of 0.17% in the total pool of fat (Figure 12). The percentage share of α-linolenic acid, EPA, and DHA in the total pool of fat extracted from each taxon oscillated between 10.1 (*S. sclarea*) and 21.0% (*S. officinalis* subsp. *lavandulifolia*), 0.4 (*S. sclarea*) and 2% (*S. officinalis* subsp. *lavandulifolia*), and 0.3 (*S. sclarea*) and 0.6% (*S. officinalis*), respectively (Figure 12).

##### Omega-6 Fatty Acids

Omega-6 acids, from the class of PUFAs, which have the first double bond located at the sixth carbon from the methyl end of the carbon chain, were the second abundant group of omega fatty acids. The highest content of these fatty acids was determined in *S. officinalis* (0.78 g·100 g^−1^ DW), an intermediate level was detected in *S. officinalis* subsp. *lavandulifolia* (0.67 g·100 g^−1^ DW), and the lowest content was found *S. sclarea* (0.24 g·100 g^−1^ DW), with a significant percentage drop by 14 and 69%, respectively (Figure 9). A similar tendency was found for the percentage share of omega-6 acids in the total pool of fats in each of the three taxa, with the value of this parameter of 14.45, 8.58, and 6.14%, respectively (Figure 10).

The following omega-6 acids were identified in the *Salviae herba* of the three analysed taxa: *Z*-11,14-eicosadienoic acid C20:2n6 (0.014 g·100 g^−1^ DW in *S. officinalis* subsp. *lavandulifolia*–0.031 g·100 g^−1^ DW in *S. sclarea*), 13,16-docosadienoic acid C22:2n6 (0.008 in *S. officinalis* and *S. sclarea*–0.015 in *S. officinalis* subsp. *lavandulifolia*), DGLA Dihomo-γ-linolenic acid 20:3n6 (0.003 g·100 g^−1^ DW in *S. sclarea*–0.012 g·100 g^−1^ DW in *S. officinalis* subsp. *lavandulifolia*), and the sum of *Z*-9,12-octadecadienoic (linoleic) and *E*-9,12-octadecadienoic (linoelaidic) acids C18:2n6c + C18:2n6t (0.182 g·100 g^−1^ DW in *S. sclarea*–0.744 g·100 g^−1^ DW in *S. officinalis*) (Figure 11). The range of the percentage share of omega-6 fatty acids in the total pool of fat extracted from the individual taxa of *Salviae herba* was as follows: *Z*-11,14-eicosadienoic acid C20:2n6 from 0.18% in *S. officinalis* subsp. *lavandulifolia* to 0.8% in *S. sclarea*, *Z*-13,16-docosadienoic acid C22:2n6 from 0.15% in *S. officinalis* to 0.22% in *S. sclarea*, DGLA 20:3n6 from 0.09% in *S. sclarea* to 0.15% in *S. officinalis* subsp. *lavandulifolia*, and the sum of linoleic and linoelaidic acids C18:2n6c + C18:2n6t from 4.75% in *S. sclarea* to 13.81% in *S. officinalis* (Figure 12). Additionally, γ-linolenic acid (C18:3n6 gamma, GLA) (0.01 g·100 g^−1^ DW) and arachidonic acid (C20:4n6) (0.001 g·100 g^−1^ DW) were identified only in the herb of *S. sclarea*, with their percentage share in the total pool of fat ranging from 0.25 to 0.03%, respectively (Figure 11 and Figure 12). Omega-3 and omega 6-acids, contrary to mono-unsaturated omega-9, constitute a group of essential polyunsaturated fatty acids (EFAs), which cannot be synthesised in the human organism.

##### Omega-9 Fatty Acids

Among the three examined taxa, the *S. officinalis* subsp. *lavandulifolia* herb was characterised by the highest content (0.50 g·100 g^−1^ DW) of omega-9 fatty acids with one double bond located at the ninth carbon from the methyl end of the carbon chain, followed by a moderate level of these acids detected in *S. officinalis* (0.37 g·100 g^−1^ DW), and the lowest content in *S. sclarea* (0.21 g·100 g^−1^ DW), with a significant drop by approximately 26 and 58%, respectively (Figure 9). The percentage share of omega 9 acids in the total pool of fats was quite similar in the herb of *S. officinalis* (6.9%) and *S. officinalis* subsp. *lavandulifolia* (6.4%) and markedly higher than in *S. sclarea* (5.5%) (Figure 10).

Considering the qualitative analysis of the composition of omega-9 fatty acids, the sum of oleic and elaidic acids (C18:1n9c + C18:1n9t) as well as eruic acid (C22:1n9) were identified in all the examined *Salviae herba* samples, and their content, with the lowest value in *S. sclarea* and the highest in *S. officinalis* subsp. *lavandulifolia*, was in the range from 0.204 to 0.488 g·100 g^−1^ and from 0.002 to 0.012 g·100 g^−1^ DW, respectively (Figure 11). In *S. sclarea*, the family of omega 9 acids was also represented by nervonic acid C24:1n9, with the content of 0.005 g·100 g^−1^ DW and the percentage share in the total pool of fat 0.14% (Figure 11 and Figure 12). The range of the percentage share of the sum of oleic and elaidic acids (C18:1n9c + C18:1n9t) as well as eruic acid (C22:1n9) in the total pool of fat ranged from 5.32% (*S. sclarea*) to 6.82% (*S. officinalis*) and from 0.04% (*S. sclarea*) to 0.15% (*S. officinalis* subsp. *lavandulifolia*) (Figure 12).

##### Omega-5 Fatty Acids

In addition to the three main groups of fatty acids mentioned above, one fatty acid from the omega-5 family, i.e., myristoleic acid *Z*-9-tetradecenoic acid (C14:1n5), was detected in the samples all of the three *Salviae herba* types at concentrations between 0.002 (*S. sclarea*) and 0.012 g·100 g^−1^ DW (*S. officinalis* subsp. *lavandulifolia*) and the range of the percentage share in the total pool of fat from 0.06% to 0.15% (Figure 11 and Figure 12). Another omega-5 fatty acid, *Z*-10-pentadecenoic acid (C15:1n5), was identified only in the herb of *S. sclarea* (0.51 g·100 g^−1^ DW) (Figure 11), with the percentage share in the total pool of fat 0.51% (Figure 12).

##### Omega-7 Fatty Acids

Palmitoleic acid (C16:1n7) and *Z*-10-heptadecenoic acid (C17:1n7) from the omega-7 family were detected in the analysed material with the level ranging from 0.014 g·100 g^−1^ DW (*S. sclarea*) to 0.029 g·100 g^−1^ DW (*S. officinalis*) and from 0.010 (*S. officinalis*) to 0.06 g·100 g^−1^ DW (*S. sclarea*), respectively (Figure 11). The percentage share of these acids in the total pool of fat was in the range from 0.29% (*S. officinalis* subsp. *lavandulifolia*) to 0.54% (*S. officinalis*) and from 0.19% (*S. officinalis*) to 1.56% (*S. sclarea*), respectively (Figure 12).

#### 2.3.3. Qualitative and Quantitative Composition of Fatty Acids

##### Saturated Fatty Acid (SFAs)

The analyses showed the presence of 14 saturated fatty acids in the herb of *S. officinalis* subsp. *lavandulifolia* and *S. officinalis* and 15 SFAs in *S. sclarea*. The SFAs were dominated by palmitic acid (C16:0), stearic acid (C18:0), myristic acid (C14:0), arachidic acid (C20:0), and lignoceric acid (C24:0). The content of these acids was within the range of 0.759–1.152 g·100 g^−1^ DW, 0.17–0.25 g·100 g^−1^ DW, 0.15–0.17 g·100 g^−1^ DW, 0.026–0.143 g·100 g^−1^ DW, and 0.017–0.094 g·100 g^−1^ DW, respectively. The highest content of palmitic, arachidic, and lignoceric acids was found in *S. officinalis* subsp. *lavandulifolia*, while the lowest level was detected in *S. sclarea*, with statistically confirmed changes between the three taxa. The level of stearic acid was notably lower in *S. officinalis* than in *S. officinalis* subsp. *lavandulifolia* and *S. sclarea*. In turn, the myristic acid content in the *S. officinalis* and *S. sclarea* herb highly exceeded that found in *S. officinalis* subsp. *lavandulifolia* (Figure 11). The percentage share of the content of palmitic acid (C16:0), stearic acid (C18:0), myristic acid (C14:0), arachidic acid (C20:0), and lignoceric acid (C24:0) in the total pool of fat extracted from each taxon of *Salviae herba* was 14.8–19.8%, 3.2–6.3%, 1.9–4.5%, 0.7–1.8%, and 0.4–1.2%, respectively. The lowest percentage share of palmitic and myristic acids in the total pool of fat was shown in the *S. officinalis* subsp. *lavandulifolia* herb, while the highest level was found in *S. sclarea*. The opposite tendency was recorded in the case of arachidic and lignoceric acids. In turn, the value of this parameter for stearic acid in *S. officinalis* subsp. *lavandulifolia* and *S. officinalis* was markedly lower than in *S. sclarea* (Figure 12). The *S. officinalis* subsp. *lavandulifolia* herb was characterised by extremely higher content of undecanoic acid C11:0 and its high share in the total pool of fat (0.187 g·100 g^−1^ DW g and 2.40%), compared to *S. officinalis* (0.0065 mg·100 g^−1^ and 0.12%) and *S. sclarea* (0.001 g·100 g^−1^ DW and 0.03%) (Figure 11 and Figure 12). In the group of saturated fatty acids present in the other taxa, the *S. officinalis* subsp. *lavandulifolia* herb did not contain heneicosylic (C21:0) and tricosanoic (C23:0) acids. Tridecanoic acid (C13:0) and heneicosylic acid were not detected in *S. officinalis*, and no tricosanoic acid was found in *S. sclarea* (Figure 11 and Figure 12).

##### Mono-Unsaturated (Monoenoic) Fatty Acids

Six monoenoic acids were detected in the first two taxa, and eight acids from this group were found in *S. sclarea*. The highest levels were determined in the case of the sum of oleic and elaidic acids C18:1n9c+C18:1n9t (0.204 g·100 g^−1^ DW in *S. sclarea*–0.488 g·100 g^−1^ DW in *S. officinalis* subsp. *lavandulifolia*), *Z*-10-heptadecenoic acid C17:1n7 (0.01 in *S. officinalis* –0.06 g·100 g^−1^ DW in *S. sclarea*), palmitoleic acid C16:1n-7 (0.014 g·100 g^−1^ DW in *S. sclarea*–0.029 g·100 g^−1^ DW in *S. officinalis*), and *Z*-5-eicosenoic acid C20:1n15 (0.011 in *S. officinalis*–0.019 in *S. sclarea* and *S. officinalis* subsp. *lavandulifolia*), with the following range of the percentage share of these acids in the total pool of fat: 5.32–6.25%, 0.19–1.56%, 0.29–0.54%, and 0.20–0.49% (Figure 11 and Figure 12). The values of the sum of oleic and elaidic acids as well as *Z*-10-heptadecenoic acid and palmitoleic acid varied significantly between the three taxa, and the content of *Z-5-eicosenoic acid* in *S. officinalis* subsp. *lavandulifolia* and *S. sclarea* markedly exceeded the values found in *S. officinalis*. Among the monounsaturated fatty acids that were detected in *S. sclarea*, 10-pentadecenoic acid (C24:1n9) and nervonic acid (C15:1n5) were absent in the herb of *S. officinalis* subsp. *lavandulifolia* and *S. officinalis* (Figure 11 and Figure 12).

##### Two-Unsaturated (Dienoic) Fatty Acids

Three dienoic acids were detected, with the highest concentrations of the sum of linoleic and linoelaidic acids C18:2n6c + C18:2n6t (0.182 g·100 g^−1^ DW in *S. sclarea*–0.744 g·100 g^−1^ DW in *S. officinalis*), an intermediate level of *Z*-11,14-eicosadienoic acid C20:2n6 (0.014 in *S. officinalis* subsp. *lavandulifolia*–0.031 g·100 g^−1^ DW in *S. sclarea*), and the lowest content of 13,16-docosadienoic acid C22:2n6 (0.008 g·100 g^−1^ in *S. officinalis* and *S. sclarea*–0.015 g·100 g^−1^ DW in *S. officinalis* subsp. *lavandulifolia*), with the following range of the percentage share of these acids in the total pool of fat: from 4.8% (*S. sclarea*) to 13.8% (*S. officinalis*), from 0.18% (*S. officinalis* subsp. *lavandulifolia*) to 0.8% (*S. sclarea*), and from 0.15% (*S. officinalis*) to 0.22% (*S. sclarea*) (Figure 11 and Figure 12).

##### Tri-Unsaturated (Trienoic) Fatty Acids

Additionally, four trienoic acids with a dominance of α-linolenic acid (C18:3n3 alpha) (10–20%) were identified. In each of the three examined taxa, α-linolenic acid C18:3n3 alpha and Dihomo-γ-linolenic acid (DGLA) 20:3n6 were detected with the content between 0.387 g·100 g^−1^ DW (*S. sclarea*) and 1.634 g·100 g^−1^ (*S. officinalis* subsp. *lavandulifolia*) and from 0.003 (*S. sclarea*) to 0.012 g·100 g^−1^ DW (*S. officinalis* subsp. *lavandulifolia*), respectively. The range of the percentage share of these acids in the total pool of fat was 10.07–20.95% and 0.09–0.15%. Among trienoic acids present in *S. sclarea*, no γ-linolenic acid (C18:3n6, GLA) was detected in the herb of *S. officinalis* subsp. *lavandulifolia* and *S. officinalis*, while *Z*-11,14,17-eicosatrienoic acid (C20:3n3) was not contained in *S. officinalis* and *S. sclarea* (Figure 11 and Figure 12).

##### Four- Five and Six-Unsaturated (Tetra-, Penta-, and Hexanoic) Acids

Arachidonic acid C20:4n6, which represents tetraenoic acids, was identified only in *S. officinalis* (0.001 g·100 g^−1^ DW), while pentaenoic timnodonic acid EPA (C20:5n3) with the range from 0.014 g·100 g^−1^ (*S. sclarea*) to 0.153 g·100 g^−1^ DW (*S. officinalis* subsp. *lavandulifolia*) and hexaenoic DHA cervonic acid (C22:6n3) with the range from 0.013 g·100 g^−1^ DW (*S. sclarea*) to 0.037 g·100 g^−1^ DW (*S. officinalis* subsp. *lavandulifolia*) were detected in all the three analysed taxa (Figure 11). The ranges of the percentage share of the two aforementioned acids in the total pool of fat extracted from each taxon of *Salviae herba* were 0.37–1.96% and 0.33–0.57% (Figure 11 and Figure 12).

The nomenclature, abbreviations, symbols, and linear and structural formulas of the SFAs, MUFAs, and PUFAs identified in the *Salviae herba* are presented in Table 2, Table 3 and Table 4.

### 2.4. Vitamin C

**Figure 13:**
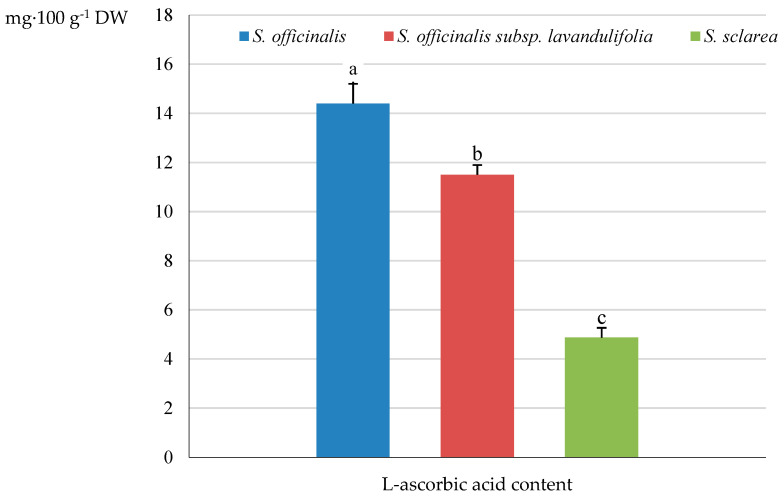
Content of vitamin C in *Salviae herba* samples. Notes: Means followed by the same letter do not differ at a significance level of α = 0.05. Vertical bars represent the standard deviations of the means.

The highest content of L-ascorbic acid from the group of unsaturated polyhydroxy alcohols was determined in the herb of *S. officinalis* (14.43 mg·100 g^−1^ DW), followed by an approximately 21% lower level in *S. officinalis* subsp. *lavandulifolia* (11.52 mg·100 g^−1^ DW) and a 66% lower value in *S. sclarea* (4.87 mg·100 g^−1^ DW) (Figure 13).

### 2.5. Polyphenolic Compounds

Polyphenolic compounds include very diverse groups in terms of biological functions, properties, and structure: phenolic acids, lignans, flavonoids (flavonols, flavanols, flavones, isoflavones, flavanones, anthocyanins, and chalcones), xanthones, anthraquinones, stilbenes, naphthoquinones, tannins, nitrogen compounds, and terpenoids.

#### 2.5.1. Cinnamic Acid Derivates

**Figure 14:**
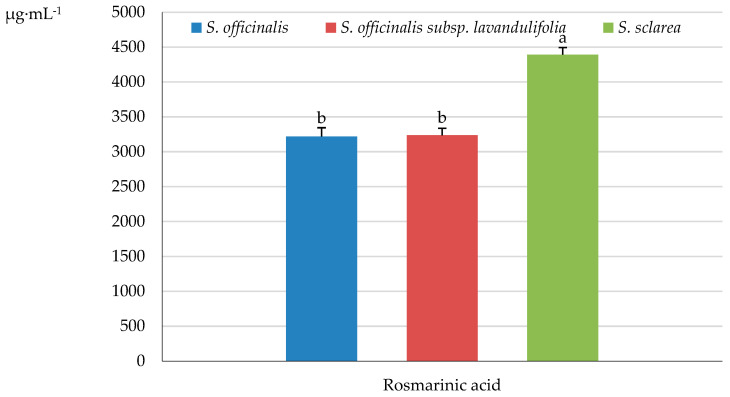
Content of rosmarinic acid in *Salviae herba* samples. Notes: Means followed by the same letter do not differ at a significance level of α = 0.05. Vertical bars represent the standard deviations of the means.

**Figure 15:**
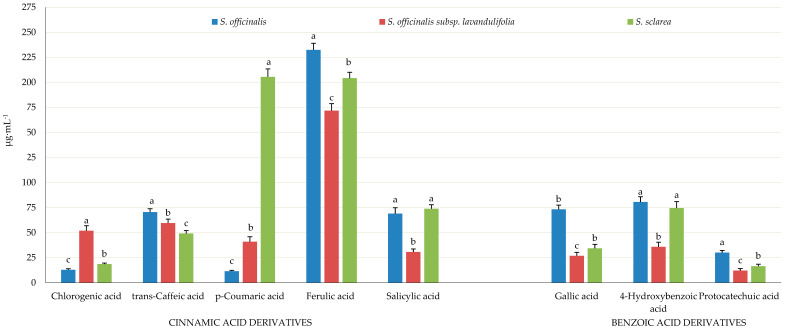
Content of other selected phenolic compounds in *Salviae herba* samples. Notes: For each compound, means followed by the same letter do not differ at a significance level of α = 0.05. Vertical bars represent the standard deviations of the means.

Rosmarinic acid was recognised as the most abundant phenolic compound in the *Salvia* samples (stem + leaves + inflorescence), with the highest level of this frequently occurring ester of caffeic acid and 3,4-dihydroxyphenyl lactic acid found in *S. sclarea* (4392.06 μg·mL^−1^), moderate amounts determined in *S. officinalis* subsp. *lavandulifolia* (3236.63 μg·mL^−1^), and the lowest content detected in *S. officinalis* (3218.87 μg·mL^−1^) (Figure 14 and Figure 15).

The content of the most common hydroxy derivatives of cinnamic acid, i.e., *p*-coumaric acid (4-hydroxycinnamic acid, 4-HCA), caffeic acid (3,4-dihydroxycinnamic acid, 3,4-dHCA), and ferulic acid (4-hydroxy-3-methoxycinnamic acid), differed notably between the examined taxa and oscillated from 11.57 (*S. officinalis*) to 205.53 μg·mL^−1^ (*S. sclarea*), from 49.16 (*S. sclarea*) to 70.53 μg·mL^−1^ (*S. officinalis*), and from 171.78 (*S. officinalis* subsp. *lavandulifolia*) to 232.43 μg·mL^−1^(*S. officinalis*), respectively. The lowest content of chlorogenic acid, also known as 5-*O*-caffeoylquinic acid, which is an ester formed from cinnamic acids and quinic acid was found in *S. officinalis* (12.83 μg·mL^−1^), followed by a markedly increased level in *S. sclarea* (18.6 μg·mL^−1^) and *S. officinalis* subsp. *lavandulifolia* (51.92 μg·mL^−1^). The level of salicylic acid synthesised from *E*-cinnamic acid by decarboxylation to benzoic acid and further 2-hydroxylation of benzoic acid was similar in *S. officinalis* and *S. sclarea* (69.07 and 73.93 μg·mL^−1^, respectively) and notably exceeded the content determined in *S. officinalis* subsp. *lavandulifolia* (30.95 μg·mL^−1^) (Figure 15).

#### 2.5.2. Benzoic Acid Derivatives

The amount of gallic acid (3,4,5-trihydroxybenzoic acid) and 4-hydroxybenzoic acid in the *Salvia* samples (stem + leaves + inflorescence) was the lowest in *S. officinalis* (26.93 and 35.81 μg·mL^−1^, respectively), moderate in *S. sclarea* (34.32 and 74.51 μg·mL^−1^), and the highest in *S. officinalis* subsp. *lavandulifolia* (73.32 and 80.67 μg·mL^−1^), while the content of protocatechuic acid (3,4-dihydroxybenzoic) exhibited the following order: *S. officinalis* (30.15 μg·mL^−1^) > *S. sclarea* (16.56 μg·mL^−1^) > *S. officinalis* subsp. *lavandulifolia* (12.17 μg·mL^−1^) (Figure 15).

### 2.6. Coumarins and Phenol Aldehyde

**Figure 16:**
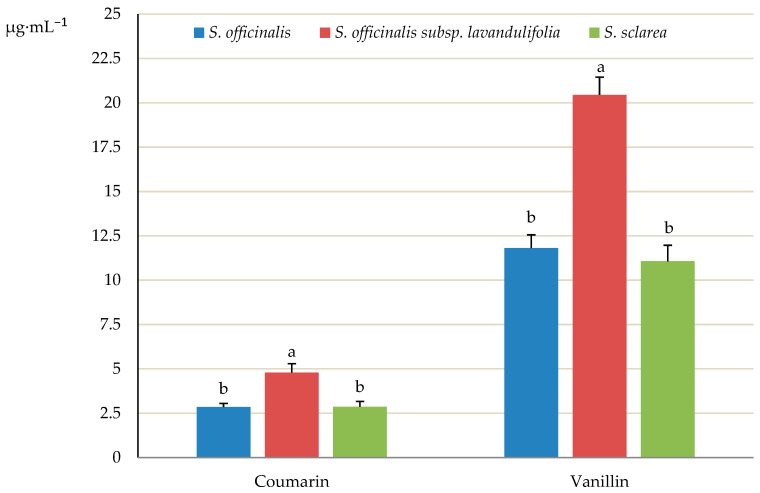
Content of coumarin and vanillin in *Salviae* samples. Notes: Means followed by the same letter do not differ at a significance level of α = 0.05. Vertical bars represent the standard deviations of the means.

The levels of the phenolic aldehyde vanillin (4-hydroxy-3-methoxybenzaldehyde) and coumarin (1-Benzopyran-2-one) were quite similar in *S. officinalis* (11.82 and 2.85 μg·mL^−1^, respectively) and *S. sclarea* (11.07 and 2.87 μg·mL^−1^) and lower than in *S. officinalis* subsp. *lavandulifolia* (20.45 and 4.79 μg·mL^−1^) (Figure 16).

### 2.7. Non-Phenolic Organic Acids

**Figure 17:**
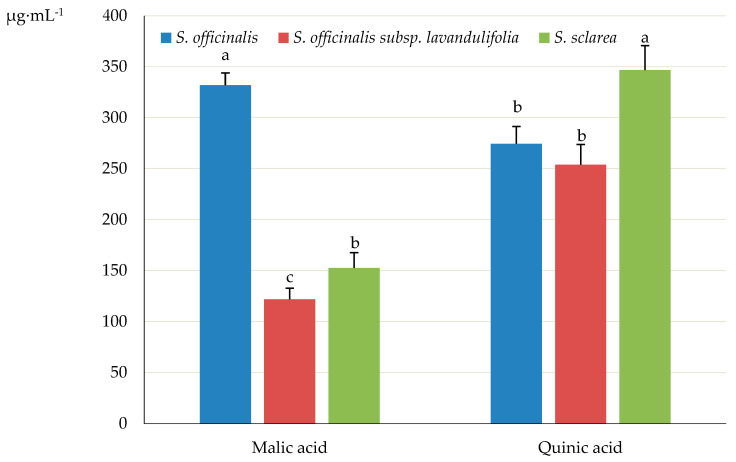
Content of selected non-phenolic organic acids in *Salviae* samples. Notes: Means followed by the same letter do not differ at a significance level of α = 0.05. Vertical bars represent the standard deviations of the means.

The content of malic and quinic acids in the *Salvia* samples (stem + leaves + inflorescence), ranging from 121.89 (*S. officinalis* subsp. *lavandulifolia*) to 331.95 (*S. officinalis*) μg·mL^−1^ and from 253.84 (*S. officinalis* subsp. *lavandulifolia*) to 346.69 μg·mL^−1^ (*S. sclarea*), differed significantly between the three taxa (Figure 17).

### 2.8. Ortho-Dihydroxyphenols

**Figure 18:**
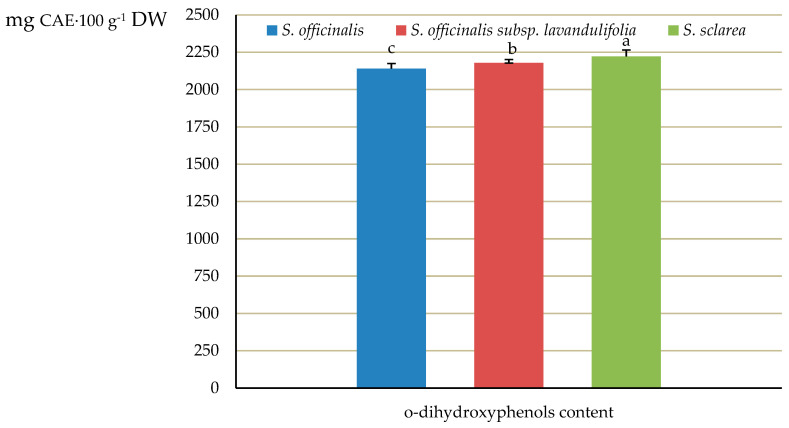
Content of *o*-dihydroxyphenols in *Salviae herba* samples (calculated as caffeic acid equivalents, CAE). Notes: Means followed by the same letter do not differ at a significance level of α = 0.05. Vertical bars represent the standard deviations of the means.

The total levels of *o*-dihydroxyphenols in the analysed *Salviae herba* samples varied notably between the three analysed taxa, with the highest concentration in *S. sclarea* (2222 mg·100 g^−1^ DW calculated as caffeic acid equivalents, CAE), an intermediate level in *S. officinalis* subsp. *lavandulifolia* (2179 mg·100 g^−1^ DW), and the lowest content in *S. officinalis* (2140 mg·100 g^−1^ DW) (Figure 18).

### 2.9. Flavonoids

**Figure 19:**
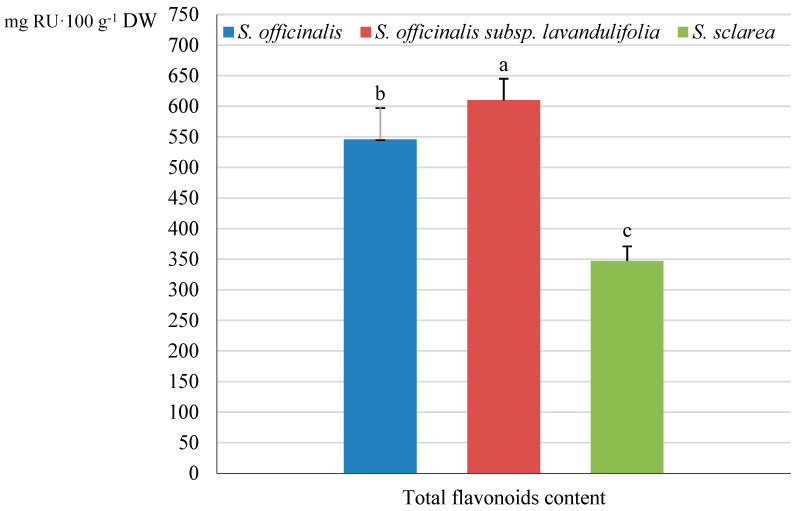
Total flavonoid content in *Salviae herba* samples (calculated as rutin equivalents). Notes: Means followed by the same letter do not differ at a significance level of α = 0.05. Vertical bars represent the standard deviations of the means.

The highest flavonoid level in the analysed *Salviae herba* was detected in *S. officinalis* subsp. *lavandulifolia* (610 mg·100 g^−1^ calculated as rutin equivalents), with a considerably lower value of this parameter by approximately 10.5% in *S. officinalis* (546 mg RU·100 g^−1^ DW) and by 43% in *S. sclarea* (347 mg RU·100 g^−1^ DW) (Figure 19).

#### 2.9.1. Flavones

**Figure 20:**
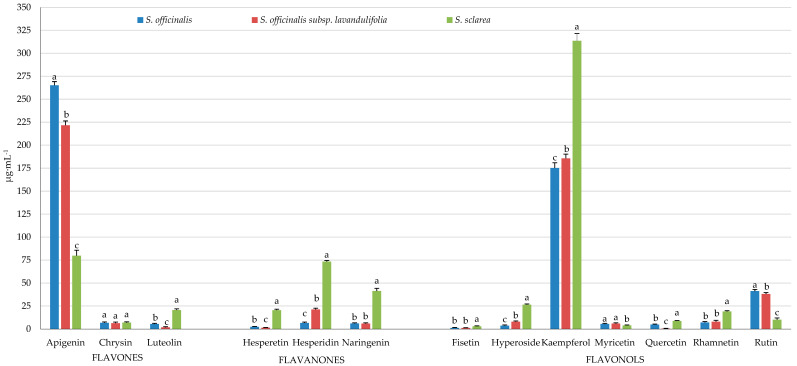
Content of selected flavonoids in *Salviae herba* samples. Notes: For each flavonoid, means followed by the same letter do not differ at a significance level of α = 0.05. Vertical bars represent the standard deviations of the means.

The dominant flavone in the samples (stem + leaves + inflorescence) of the analysed taxa was apigenin, with the highest level in *S. officinalis* (265 μg·mL^−1^), followed by *S. officinalis* subsp. *lavandulifolia* (222 μg·mL^−1^) and *S. sclarea* (80 μg·mL^−1^). The luteolin content increased notably in the following order: *S. officinalis* subsp. *lavandulifolia* (1.82 μg·mL^−1^) < *S. officinalis* (5.39 μg·mL^−1^) < *S. sclarea* (20.60 μg·mL^−1^). The chrysin content in the examined taxa was similar and oscillated within 6.23–6.89 μg·mL^−1^ (Figure 20).

#### 2.9.2. Flavanones

The samples of *S. sclarea* (stem + leaves + inflorescence) were the richest source of flavanones of the three analysed taxa. The range of hesperetin and hesperidin content fluctuated between 1.39 (*S. officinalis* subsp. *lavandulifolia*) and 20.38 μg·mL^−1^ DW (*S. sclarea*) and from 6.58 (*S. officinalis*) to 73.35 μg·mL^−1^ DW (*S. sclarea*), respectively. The level of naringenin in *S. officinalis* subsp. *lavandulifolia* and *S. officinalis* was comparable (approximately 6.00 μg·mL^−1^ DW), but it was distinctly lower than in *S. sclarea* (41.30 μg·mL^−1^ DW) (Figure 20).

#### 2.9.3. Flavonols

Kaempferol and rutin were the dominant flavonols in the samples (stem + leaves + inflorescence) of the analysed three taxa, with the content range from 175.32 (*S. officinalis*) to 313.64 μg·mL^−1^ (*S. sclarea*) and from 10.03 (*S. sclarea*) to 41.17 (*S. officinalis*), respectively. Fisetin was recognised as the most abundant flavonol, with the level ranging between 1.11 (*S. officinalis* subsp. *lavandulifolia*) and 2.89 μg·mL^−1^ (*S. sclarea*). The content range of the other flavonols in the of the examined taxa was as follows (in μg·mL^−1^): hyperoside from 3.57 (*S. officinalis*) to 26.37 (*S. sclarea*), rhamnetin from 7.04 (*S. officinalis*) to 19.04 (*S. sclarea*), quercetin from 0.46 (*S. officinalis* subsp. *lavandulifolia*) to 8.76 (*S. sclarea*), and myricetin from 3.83 (*S. sclarea*) to 5.86 μg·mL^−1^ (*S. officinalis* subsp. *lavandulifolia*). Therefore, the highest level of flavonols, except for rutin and myricetin, was found in *S. sclarea*, which contained the lowest level of these two flavonols (Figure 20).

## 3. Discussion

### 3.1. Ash

The ash content in the *S. officinalis* herb (9.24% DW) determined in the present study was similar to that in the aboveground parts of this species described in the literature (8.76; 9.10% DW) [66,67] and leaves (9.10; 9.87; 12.59%) [66,68,69], but it was higher than its content in *Salviae folium* from common sage (2.17% DW) reported by [70]. The ash content determined in *S. sclarea* (11.9% DW) in the present study was lower than that reported in *Salviae herba* (18.39% DW) from this species [67] and similar to the content in *Salvae folium* (12.59% DW) [68]. Simultaneously, the ash content in the herb of the three taxa from the genus *Salvia* exceeded the value of this parameter reported by other authors *analysing Salviae hispanicae semen* (chia seeds) (7.7% DW) [71].

### 3.2. Protein

In the present study, the protein content in the *S. sclarea* herb (8.57%) was lower than that reported in the literature for this species (14.49% DW) [67]. The total protein content in the *S. officinalis* herb (16.14% DW) obtained in this study was higher than the amounts detected in its aboveground parts (6.86–7.13, 9.38, and 15.28% DW) by other authors [67,69,72], but higher (17.07%) and lower (15.28% DW) than that recorded in the leaves of this species by Darwish et al. [68] and Draz et al. [70], respectively. The three *Salvia* taxa analysed in the present study exhibited significantly higher crude protein content in the herb than the *S. hispanica* species (5.73 g·100 g^−1^ DW) studied by Peiretti and Gai [71].

As shown by Grancieri et al. [73] protein isolated from *S. hispanica* seeds and protein fractions: albumin, globulin, glutelin, and prolamin digested in simulated gastrointestinal conditions in the inflammatory process reduced p-NF-κB (phospho-nuclear factor kappa B), iNOS (inducible nitric oxide synthase enzyme), p-JNK (phospho c-Jun N-terminal kinases), and AP-1, which is a sequence-specific transcriptional activator composed of members of the Jun and Fos families. They also reduced the NF-κB translocation to nuclei. Furthermore, protein and glutein inhibited the production of prostaglandins, TNF-α (tumour necrosis factor α), MCP-1 (monocyte chemoattractant protein-1), IL-6 and IL-10, nitric oxide release, reduced the generation of reactive oxygen species, NF-κB translocation to nuclei, and the expression of iCAM (intercellular adhesion molecule 1) and LOX-1 (low-density lipoprotein receptor-1), and limited lipid accumulation. As a result, protein fractions limited the levels of markers involved in the induction of pro-inflammatory processes and atherosclerosis in macrophages (in silico, in vitro) [73]. Albumin and globulin peptides from *S. hispanica* seeds exhibited strong antiradical activity against 2,2-diphenyl-1-picrylhydrazyl (DPPH) and 2,2′-azino-bis(3-ethylbenzothiazoline-6-sulphonic acid) (ABTS) and inhibited the angiotensin-converting enzyme (ACE; EC 3.4.15.1) activity to a great extent. These fractions exerted strong chelation ability [74]. Furthermore, lectin isolated from *S. sclarea* seeds recognised the abnormal *O*-glycan called the Tn antigen defined as the monosaccharide N-acetylgalactosamine linked to serine or threonine GalNAca-O-Ser/Thr, which is a specific marker in many human carcinomas [75].

### 3.3. Amino Acids

Amino acids play a key role in the successful cultivation of medicinal and aromatic plants, especially under environmental stress [76]. They are used by plants for many different purposes, including the synthesis of substances with high biological activity, the generation of energy, and the biosynthesis of proteins [77]. Approximately 20 amino acids are involved in early stages of each protein synthesis in a cell [76,77], who investigated growth characteristics and changes in the active ingredients of *Salvia mirzayanii* essential oil, have suggested that foliar application of amino acids stimulates metabolic processes, thus improving quantitative and qualitative yield. Amino acids directly regulate vital function and plant structure. They are recognised as biostimulants crucial in the synthesis of secondary metabolites, including hormone-derived ones. Amino acids in plants enhance mRNA transcription, activate hormones responsible for reproductive growth, stimulate the formation of chlorophyll, carotenoids, and carbohydrate, improve the essential oil profile, and increase the uptake and transport of elements [78].

#### 3.3.1. Exogenous Amino Acids

In the present study, eight protein amino acids were classified as exogenous. Their content ranged from 26.05 mg·g^−1^ DW (*S. sclarea*) to 52.27 mg·g^−1^ DW (*S. officinalis*), which constituted 41–47% of the total amino acid pool. Leucine: 5.06 mg·g^−1^ (*S. sclarea*)–10.1 mg·g^−1^ (*S. officinalis*), phenylalanine: 3.87 mg·g^−1^ (*S. sclarea*)–8.25 mg·g^−1^ (*S. officinalis*), and lysine: 3.34 (*S. sclarea*)–6.67 mg·g^−1^ (*S. officinalis*) were the predominant amino acids. This is consistent with the results reported by Laftouhi et al. [72], who found the highest amounts of leucine and phenylalanine in *S. officinalis* leaves, and with the investigations of lectin proteins in *S. sclarea* seeds conducted by Medeiros et al. [75], in which phenylalanine and leucine ranked second and fourth, respectively. Darwish et al. [68] reported the dominance of exogenous amino acids: lysine, phenylalanine + tyrosine, leucine, and valine in *S. officinalis* leaves. A study conducted by Koshevoi [79] indicated phenylalanine, isoleucine, and leucine as the dominant exogenous amino acids in *S. officinalis* leaves. The profile of exogenous amino acids determined in the present study is consistent with the results reported by Vovk et al. [80]. These researchers reported the highest concentrations of leucine, phenylalanine, and isoleucine in *Salvia flos*. Leucine was the dominant exogenous amino acid in the three taxa of the *Salvia* genus analysed in the present study. Supplementation with leucine-rich protein is one of the methods for preventing sarcopenia [81]. It also exerts a beneficial effect on muscle strength, volume, and function and reduces inflammation, ultimately improving the health status in patients with cerebral palsy [82]. Furthermore, leucine increases lean body mass [83]. The anticatalytic effect of leucine is a consequence of the intra-systemic synthesis of beta-hydroxy-β-methylbutyrate in skeletal muscle and liver, which inhibits protein degradation in skeletal muscles. In healthy individuals, approximately 60% of dietary leucine is metabolised after several hours, with around 5% (2–10% range) being converted to HMB [84,85]. Leucine also exerts an anabolic effect, supporting muscle tissue growth through activation of the mTOR (mammalian target of rapamycin) signalling pathway responsible for initiating muscle protein synthesis, with the synergistic participation of the AMP-activated protein kinase (AMPK) catabolic signalling pathway that coordinates cell growth, autophagy, and metabolism [86,87,88]. Human muscle cells sense a rise in leucine and/or essential amino acids to trigger mTOR activation with the involvement of hVps34 and MAP43K kinases [89,90]. Leucine sensor leucyl-tRNA synthetase (LARS) and stress response protein 2 (Sestrin2) are involved in the leucine-sensing process and the activation of RagB/RagD [91].

Phenylalanine, the second most abundant amino acid identified in *Salvia* herb in the present study, is a precursor of neurotransmitters (adrenaline, dopamine), enzymes, and hormones (thyroxine) [92,93,94,95]. It exhibits neurological and dermatological benefits and is crucial for melanin synthesis. It was revealed that amino acid transporter SLC16A10 enhances melanogenesis by promoting the uptake of phenylalanine, and upregulation SLC16A10 is likely responsible for melasma and the UVB-induced hyperpigmentation [96]. L-phenylalanine administered orally or topically to the skin, in combination with exposure to UVA light, alleviates the symptoms of vitiligo, regulates melanogenesis, inhibits tyrosinase activity, and consequently reduces serum levels of tyrosine, which limits its uptake [97]. Upon conversion into L-tyrosine with the involvement of the enzyme phenylalanine hydroxylase (PAH) and its cofactor tetrahydrobiopterin, phenylalanine is involved in the synthesis of the noradrenaline and dopamine crucial for the proper functioning of the brain and the nervous system [92,93]. Phenylalanine is recommended in the treatment of some types of depression, schizophrenia, and Parkinson’s disease [98,99,100,101,102]. The metabolism pathway of this amino acid (increased Phe levels in blood) seems to be the most important pathway in attention deficit hyperactivity disorder (ADHD) [95]. Phe antagonises G-protein coupled receptors for gamma-aminobutyric acid (GABAB receptors) involved in analgesia, cardiovascular regulation, and depression, suppressing their inhibitory effects [103,104]. Phenylalanine is an ingredient of some artificial sweeteners i.e., aspartame (L-aspartyl-L-phenylalanine methyl ester). It tends to decrease appetite [105].

In the present study, the content of valine, i.e., another exogenous amino acid, in the herb of the analysed taxa ranked third in *S. sclarea* (3.39 mg·g^−1^ DW) and fourth in *S. officinalis* subsp. *lavandulifolia* (5.52 mg·g^−1^ DW) and *S. officinalis* (6.59 mg·g^−1^ DW). This is consistent with reports on this amino acid in aboveground parts of sage [72] and with the content of lectins in *S. sclarea* seeds reported elsewhere [75]. It has been shown that low blood valine levels are associated with a high risk of hip fractures [106]. The results obtained in the present study are in agreement with other reports [107]. Valine enhances the respiration rates of complex I (NADH/ubiquinone oxidoreductase), II (succinate dehydrogenase), and IV (cytochrome c oxidase), but does not change the activity of complex III (cytochrome c reductase) [108]. The role of this amino acid in regulating complex I is well documented in T-cell acute lymphoblastic leukaemia [109]. Valine treatment improved the oxygen consumption rate (OCR) and the extracellular acidification rate (ECAR) in the C2C12 mouse cell line probably as a result of extreme oxidation of fatty acids, which inactivate pyruvate dehydrogenase blocking the process of glycolysis. Branched-chain amino acids (BCAAs) are able to sustain oxidative phosphorylation and enhance ATP generation during oxidative stress; this is important for muscle tissue, which is susceptible to high risk of oxidative stress [108].

#### 3.3.2. Endogenous Amino Acids

The present results show that the group of endogenous amino acids in the *S. officinalis* and *S. officinalis* subsp. *lavandulifolia* herb was dominated (mg·g^−1^ DW) by glutamic acid (17.60 and 12.10) and aspartic acid (16.80 and 12.30, respectively), followed by alanine (7.90 and 6.49), glycine (6.32 and 5.39), proline (5.40 and 5.18), serine (5.67 and 4.70), and tyrosine (3.53 and 3.38 mg·g^−1^ DW, respectively). As shown by Laftouhi et al. [72], *S. officinalis* leaves did not contain alanine, asparagine, and glutamine when grown in normal conditions and various climatic disturbances or tyrosine in plants growing at increasing temperature and decreasing precipitation, while serine, proline, and glycine were the dominant endogenous amino acids. The content of the dominant exogenous amino acids determined by these researchers in unchanged growth conditions was 2.42 (Ser), 4.06 (Pro) and 5.54-fold (Gly) lower than that obtained in the present study and amounted to 2.34, 1.33 and 1.14 mg·g^−1^ DW; in the stress conditions, it ranged from 1.63 to 0.45, from 1.03 to 0.51, and from 0.45 to 0.12 mg·g^−1^ DW, respectively. Similarly to our studies, Myha et al. [110] recognised glutamic acid (10.8 mg·g^−1^ DW) and aspartic acid (9.7 mg·g^−1^ DW) as the dominant non-essential amino acids in *S. officinalis* leaves. The levels of Asp, Glu, Ala, Ser, Gly, and Tyr reported by these authors were 1.73, 1.63, 1.49, 1.67, 1.29, and 1.26 times lower, respectively, and the amount of Pro was 1.8-fold higher than that in the present study. In *S. officinalis* leaves, Darwish et al. [68] found that glycine (10.2), glutamic acid (8.3), alanine (7.5), and aspartic acid (5.3 mg·g^−1^ DW) were the dominant endogenous amino acids. The content of Gly was 1.6-fold higher and the level of the other amino acids was 2.10 (Glu), 1.05 (Ala), 3.17 (Asp), 2.26 (Ser), 2.34 (Tyr), and 64-fold (Pro) lower than the values determined in the present study. Other investigations demonstrated that tyrosine, serine, glutamic acid, and aspartic acid were the dominant non-essential amino acids in sage leaves [79,80]. The group of endogenous amino acids in the *S. sclarea* herb analysed in the present study was dominated by proline (10.00) and glutamic and aspartic acids (7.52 and 7.55), followed by alanine (4.20), glycine (93.32), serine (2.96), and tyrosine (1.99 mg·g^−1^ DW). As reported in the literature [75,111], in the lectin isolated from nutmeg sage, glycine was the most abundant endogenous amino acid, followed by aspartic acid, serine, alanine, glycine, and tyrosine. The least abundant was proline, which was classified as the dominant amino acid in the herb of this species in the present study. Except for aspartic acid and serine in *S. officinalis* and proline in *S. sclarea* and *S. officinalis*, the content of endogenous amino acids in the herb of the three *Salvia* taxa analysed in this study was lower than that reported by Myha et al. [110] in *Salvia grandiflora*, who recognised glutamic acid (18.4) and aspartic acid (14.4) as the dominant acids and reported high content of alanine (8.6), glycine (7.7), serine (5.5), proline (5.3), and tyrosine (5.2 mg·g^−1^ DW).

The results of the present study of non-essential amino acids in the herb of the *Salvia* taxa are also consistent with the report by Siahbalaei and Kavoosi [107], who found that aspartic acid, glycine, glutamic acid, alanine, and serine were the dominant endogenous amino acids in extracts from several plant species representing the families Lamiaceae and Apiaceae. Both exogenous and endogenous amino acids prevented the oxidation of glucose, lipids, and proteins and had antiamylase and antiglucosidase activity. These compounds are highly important due to their health-enhancing biological activity.

### 3.4. Lipids

The total fat content in the *S. sclarea* herb (3.84% DW) shown in the present study was approximately two-fold higher than that determined by other authors in aboveground parts (1.99% DW) [67] and 1.83, 2.45, and 7.65 times lower than that found in the flowers (7.04), leaves (9.42), and seeds (29.38% DW) of this species, respectively [112]. In turn, the content of total fat determined in the *S. officinalis* herb (5.39% DW) in the present study was 1.3 (4.06% DW), 1.6 (3.46% DW), and 2.8-fold (1.35% DW) higher, respectively, than the level of this component recorded by Dziadek et al. [67], Darwish et al. [68], and Laftouhi et al. [72]. In contrast, it was 1.2 (6.25% DW) and 1.6 (8.55% DW) times lower than the amounts determined in the leaves of this species by Todorova et al. [69] and Draz et al. [70]. The three *Salvia* taxa analysed in the present study were characterised by a higher crude fat level than in *S. hispanica* (1.67% DW) investigated by Dziadek et al. [67].

### 3.5. Fatty Acids

#### 3.5.1. Saturated Fatty Acids

Palmitic acid (C16:0) dominated the saturated fatty acid class. Its highest content, accounting for 19.77% of the total fatty acid pool, was recorded in *S. sclarea* (0.75 g·100 g^−1^ DW), and the lowest level of 1.15 g·100 g^−1^ DW (14.77%) was found in *S. officinalis* subsp. *lavandulifolia*. In this fatty acid class, stearic acid (C18:0) and myristic acid (C14:0) ranked second and third. This is consistent with the results reported by Kara et al. [112], who found the highest percentage of palmitic acid (16.06, 11.37, and 6.24%) and stearic acid (5.37, 6.48, and 1.86%) in *S. sclarea* leaves, flowers, and seeds, respectively, and myristic acid in leaves (4.83%) and flowers (0.86%). Similarly, the aboveground parts of *S. euphratica* var. *leiocalycina, S. euphratica* var. *euphratica,* and *S. pseudoeuphratica* exhibited the highest content of palmitic acid (16.23, 11.53, and 29.45 μg·mg^−1^, respectively) and stearic acid (12.06, 31.58, and 7.48 μg·mg^−1^, respectively) [113]. In *Salvae herba* of eleven different taxa, palmitic acid (3.80–8.66%) and stearic acid (1.22–2.90%) were the most abundant in the fatty acid pool [114]. In the human organism, palmitic acid constitutes 20–30% of total fatty acids. It is produced endogenously through lipogenesis or is supplied with food [115]. This acid has many biological functions, sometimes controversial, at the cellular level [115,116,117]. This component of cell membranes and secretory and transport lipids regulates the palmitoylation of proteins, palmitoylated signalling molecules, and palmitoylethanolamide biosynthesis [115,116,117]. In the cell, palmitic acid is converted into phospholipids, diacylglycerol, and ceramides, which activate various signalling pathways and Toll-like receptors (TLR4) via lipopolysaccharide, while the metabolic products activate various protein kinases C, endoplasmic reticulum stress, and reactive oxygen species (ROS). Consequently, palmitic acid was reported to enhance TLR4-induced signalling [118]. Additionally, it reduced the expression of the sarcoplasmic/endoplasmic reticulum calcium ATPase pump, regulating insulin resistance in human endothelial cell lines [119]. Palmitic and stearic acids induced apoptosis in granulosa cells and testicular Leydig cells, consequently modulating reproductive system abnormalities in females and males, respectively [120,121].

In the present study, the content of stearic acid (C18:0) was within the range of 3.16 (*S. officinalis*)–6.25 g·100 g^−1^ DW (*S. sclarea*). At the cellular level, this acid participates in the structure of triglycerides, waxes, and glycolipids and is responsible for membrane integrity. It was reported to regenerate damaged mitochondria and enhance the β-oxidation of fatty acids [122]. It also reduced the permeability and stiffness of the stratum corneum of the epidermis, and this property was useful for development of moisturising cosmetic formulations [123,124,125,126]. Stearic acid reduced postprandial lipaemia in humans [127,128]. It also exerted a neuroprotective effect on cortical neurons, increased the activity of antioxidant enzymes, activated PPARγ, and enhanced the synthesis of new proteins [129]. It lowered HDL levels, compared to other saturated fatty acids, and ensured a favourable ratio of total cholesterol to HDL cholesterol [130]. Additionally, it inhibited proliferation and induced apoptosis in breast cancer cells and implicated protein kinase C in the signalling cascade. Upon dietary intake, it exerted a preventive effect on breast cancer in individuals at high risk of the disease [131].

In the present study, the content of myristic acid (C14:0) ranged from 1.92 (*S. officinalis* subsp. *lavandulifolia*) to 4.47 g·100 g^−1^ DW (*S. sclarea*). This acid has been found to exert an analgesic and anti-inflammatory effect in macrophages through the involvement of IL-10 [132]. The concentration of myristic acid at birth determines the development of atopic dermatitis, and this mechanism is regulated by the *FADS3rs174449* gene and is one of the protection methods [133]. Present in amniotic fluid, colostrum, and milk, it stimulates the appetite in newborns more strongly than other fatty acids [134]. It has been evidenced that myristic acid conjugated with the MC-DA7R peptide is effective in drug delivery to glioma cells (in vitro and in vivo) [135]. It stimulated the expression of apolipoprotein CIII (ApoCIII) and APOC3 mRNA in HepG2 cells and increased the TG concentration and triglyceride-rich lipoprotein (TRL) metabolism in plasma. This indicates that limited dietary intake of myristic acid may be part of a supportive therapy in cardiovascular diseases [136].

#### 3.5.2. Unsaturated Fatty Acids

The present study showed the dominance of α-linolenic acid (C18:3n3 alpha) in the group of omega-3 fatty acids, *cis*-11,14-eicosadienoic acid (C20:2n6) in the omega-6 family, and the sum of oleic acid and elaidic acids in the omega-9 class in the herb of the three taxa. This is consistent with the results reported by Darwish et al. [68], in which α-linolenic acid (omega 3), α-linoleic acid (omega 6), and oleic acid (omega 9) dominated in *S. officinalis* leaves. The aboveground parts of other *Salvia* species exhibited the dominance of α-linolenic and linoleic acids [113,114]. α-Linoleic acid is a precursor of the synthesis of eicosapentaenoic acid (EPA, 20:5n-3) and docosahexaenoic acid (DHA, 22:6n-3); despite the low conversion rate of <8% and <4%, respectively, it has therapeutic effects in many diseases [137,138]. Omega-3 fatty acids contained in *S. sclarea* essential oil participated in the eicosanoid synthesis pathway, stabilised mood, and had an anxiolytic effect in a mouse model [139]. Omega-3 fatty acids: α-linolenic, docosahexaenoic, and eicosapentaenoic acids in food products have a protective role against metabolic disorders [140].

In the present study, the concentration of *cis*-11,14-eicosadienoic acid (C20:2n6) (omega 6) ranged from 0.18 (*S. officinalis* subsp. *Lavandulifolia*) to 0.80 g·100 g^−1^ DW (*S. sclarea*). It was found that polyunsaturated fatty acids: *cis*-11,14-eicosadienoic acid (C20:2n6), *cis*-13,16-docosadienoic acid (C22:2N6), docosatetraenoic acid (C22:4n6), γ-linolenic acid (C18:3n6 gamma, GLA), and guanosine-5′-triphosphate in the brainstem of hypertensive rats induced metabolic disorders and, consequently, reduced PUFA biosynthesis [141].

In the present study, the omega 9 fatty acid group was dominated by the sum of elaidic and oleic acids. It was found that elaidic acid increased the activity of cholesterol ester transfer protein, thus increasing the LDL cholesterol concentration and reducing the HDL cholesterol level [142]. Elaidic acid had an impact on the metastatic potential of colorectal cancer (CRC) cells, which has important implications for therapy [143]. Direct effects of OA and EA on the expression of target myokines and adipokines were reported. OA, but not EA, induced IL-6 expression in skeletal muscle cells, but inhibited resistin gene expression and increased adiponectin gene expression in adipocytes in a dose-dependent manner. EA decreased IL-15 gene expression and induced TNF-α expression in skeletal muscle cells; these are novel mechanisms by which *Z*- and *E*-monounsaturated fats may regulate systemic functions [144].

### 3.6. Ascorbic Acid

Our results indicated that, among the analysed taxa, *S. officinalis* could be considered as the best source of vitamin C; hence the suggestions of using the herb as a food supplement, especially for the elderly and vegetarians. *S. sclarea* turned out to be the poorest source of ascorbate. The ascorbic acid contents determined in the herb of the three *Salvia* taxa (*S. sclarea* 4.9 < *S. officinalis* subsp. *lavandulifolia* 11.5 < *S. officinalis* 14.4 mg·100 g^−1^ DW) were significantly lower than the range found in the leaves (40.28–51.36) and stems (20.3–24.32 mg·100 g^−1^ DW) of two sage species, i.e., *Salvia tesquicola* (dry-steppe sage) and *Salvia verticillata* (lilac sage) grown in Tatarstan and Crimea, Kazan, Russian Federation, respectively [145]. The biosynthesis of ascorbic acid takes place in photosynthetic organisms. Intensive photosynthesis corresponds with increased ascorbic acid biosynthesis, and it was found that the higher concentration of ascorbate strongly positively correlates with antioxidant activity and protective function [78,146]. Vitamin C is a well-known preeminent non-enzymatic antioxidant; the reduction of the oxidised form of vitamin E promotes its penetration through cell membranes [146]. Ascorbate is one of the principal antioxidants taken in the diet. Dietary supplementation with antioxidant compounds eliminates the adverse effects of oxidative stress, thus preventing stress-related diseases [147]. Vitamin C supports the proper functions of the nervous, circulatory, and immune systems. It is one of the most powerful antioxidants in the brain, playing a key role in its health. It is required for the synthesis of collagen, which determines the structural integrity of connective tissue membranes (meninges) surrounding the brain and blood vessels. Therefore, ascorbate not only seals and strengthens blood vessels by increasing endothelial synthesis and type IV collagen deposition, but also supports the integrity of the blood–brain barrier, which defends the brain against inflammation and toxins [148,149]. The protective role of ascorbic acid in the process of neuroinflammation and oxidative damage to neurons leading to neurological conditions, i.e., such neurodegenerative and psychiatric diseases as Parkinson’s, Alzheimer’s, and Huntington’s diseases, multiple and amyotrophic sclerosis, anxiety, depression, and schizophrenia, has been documented [150,151,152]. As reported by Orywal et al. [149] the neuroprotective role of vitamin C is also associated with the removal of beta-amyloid plaques and regulation of the concentration of metal ions in the brain, especially prevention of excessive iron accumulation, thus limiting the risk of oxidative stress. Ascorbate participates in neuron repair, promoting the formation of the extracellular matrix. The documented regulation of mood, cognitive functions, and motor control by ascorbic acid is associated with its involvement in the production of neurotransmitters: serotonin, norepinephrine, and dopamine [149]. Research results suggest that, as a cofactor for the biosynthesis of amidated opioid peptides, ascorbic acid can be safely and effectively used in adjunctive therapy for relief of acute and chronic (including cancer-related) pain in specific patient groups [153]. The effect of vitamin C on the immune system is related to increased interferon production and enhanced T and B lymphocyte activity as well as protection against oxidative stress, whose level increases during infection [154,155]. Based on these results, it may be suggested that the dietary intake of sage ensures adequate vitamin C levels and offers the associated health benefits, e.g., improvement of immunity, support for the circulatory system, reduction in the risk of neurodegenerative diseases, and enhancement of overall brain health [148,149,154].

### 3.7. O-Dihydroxyphenols

In the present study, the species were ranked as follows in terms of the total content of *o*-dihydroxyphenols (mg CAE·100 g^−1^ DW): *S. officinalis* subsp. *lavandulifolia* (2140.2) < *S. officinalis* (2178.6) < *S. sclarea* (2221.5). It is well documented that *ortho*-dihydroxy (catechol) groups on one ring and *para*-dihydroxy groups on the other ring determine their powerful antioxidant effects [156]. These groups are the most important structural features involved in the high antioxidant activity of phenolic compounds. The presence of *o*-dihydroxy groups determines the biological activity of many other compounds as well, e.g., miltirone (an abietane-type diterpene quinone), i.e., a compound from *Salvia miltiorrhiza* roots exerting antioxidant, anti-inflammatory, anti-plasmodial, anti-trypanosomal, and anti-tumour effects, atuntzensin A, the flavone luteolin, and the flavonoid 7-*O*-methyl luteolin with anti-inflammatory, antibacterial, anti-tumour, anti-oxidant, and anti-viral activity, and the flavonoid eupafolin, which is a major biologically active component extracted from *Salvia plebeian* with anticonvulsive, antinoceptive, antioxidant, anti-inflammatory, and anti-tumour properties [157,158,159,160]. Similarly, rutin (also known as rutoside), which is a flavonoid glycoside combining the flavonol quercetin and the disaccharide rutinose, and the flavonoid isoquercitrin possessing *ortho*-dihydroxy phenyl groups exhibit a number of pharmacological activities, e.g., neuroprotective, cytoprotective, anti-inflammatory, antioxidant, anticarcinogenic, vasoprotective, and cardioprotective effects [161,162].

### 3.8. Phenolic Acids

In the present study, the phenolic acids identified in the herb of the analysed taxa were dominated by rosmarinic acid (3218.87 in *S. officinalis*–4392.06 μg·mL^−1^ in *S. sclarea*). The content of this acid in samples from commercially available *S. officinalis* shoots [163] and leaves [164] was 482 and 2210 μg·g^−1^ DW, respectively. In turn the rosmarinic acid content in the aboveground parts of *S. officinalis* determined in other studies was 13,680.22–18,378.00 μg·g^−1^ DW; 30,084.65 mg·kg^−1^ [165,166], while in *S. sclarea* stems 5961 μg·g^−1^ DW [164]. In a study conducted by Shekarchi et al. [167], the content of rosmarinic acid in the aboveground parts of *S. officinalis* was 39.3 mg·g^−1^. Onder et al. [168] reported that rosmarinic acid (5.137 mg·g^−1^) was the predominant phenolic acid in the aboveground parts of *S. sclarea*.

Rosmarinic acid, a derivative of caffeic acid formed from phenylalanine via an ester bond with 3,4-dihydroxyphenyllactic acid derived from tyrosine, is one of the main secondary metabolites in the non-volatile fraction of extracts of many species of the genus *Salvia* [169,170,171,172]. The biosynthesis of rosmarinic acid in explant calli (in vitro) increased after the addition of L-phenylalanine (10.00 mg·L^−1^) and L-tyrosine (10.00 mg·L^−1^) as components to the nutrient medium. This indicated the possibility of using precursors to optimise the biosynthesis of selected secondary metabolites [173]. Rosmarinic acid is a component of many food, pharmaceutical, and cosmetic products. In addition, it has a wide range of pharmacological properties, e.g., antiviral [174], antibacterial [175,176], anti-inflammatory [177,178], antidiabetic [179,180,181], antiapoptotic [182,183], and anti-inflammatory [184,185] effects. It also exhibits hepatoprotective [186], neuroprotective [187,188], chemopreventive [189], anticancer [184,190,191], and antioxidant [192,193,194] activity. Given its high antioxidant capacity, rosmarinic acid has been used as a nutraceutical compound in the food industry [171] and a therapeutic agent in many diseases. It exerted antipruritic effects and alleviated inflammation in allergic contact dermatitis in mice through inhibition of the mast cell-dependent MRGPRX2/PLCγ1 signalling pathway. This indicates that the acid can be used in the treatment of pruritus and skin inflammation [195]. Additionally, rosmarinic acid alleviated atopic dermatitis symptoms via suppression of IKK-β [196]. It reduced skin inflammation and pruritus and regulated the MRGPRX2-PLCγ1-PKC-NF-κB signalling pathway and the expression of the MRGPRX2 receptor, thereby alleviating symptoms in ACD patients [195]. Furthermore, rosmarinic acid limited oxidative stress and inflammation through activation of the Nrf2/HO-1 signalling pathway in keratinocytes and reduced the serum level of inflammatory markers, mast cell infiltration, IFN-γ/TNF-α-induced chemokine production in keratinocytes, and the level of inflammatory cytokines [197]. The anticancer activity of rosmarinic acid resulted from the activation of apoptotic pathways and inhibition of the MMP-2 and MMP-9 enzymes involved in metastasis. It reduced the expression of NF-κB and STAT3 pro-inflammatory pathways, thus preventing cancer progression and attenuating oxidative damage. It also inhibited cancer cell proliferation, induced apoptosis, and prevented metastasis in various types of cancers. This confirms that rosmarinic acid can be used in the prophylaxis and complementary therapy of cancer in combination with conventional treatment methods [198].

In the present study, ferulic acid (4-hydroxy-3-methoxy cinnamic acid) ranked second among the phenolic acids detected in the three taxa: *S. officinalis* (232.43 μg·mL^−1^), *S. officinalis* subsp. *lavandulifolia* (171.78 μg·mL^−1^), and *S. sclarea* (204.17 μg·mL^−1^). The reported in the literature content of this acid in the aboveground parts of *S. officinalis* [165] was 312.43 μg·g^−1^, while in *S. sclarea* herb 60.80 and 126.00 μg·g^−1^ [199,200]. Ferulic acid has antiviral [201,202], antibacterial [203], antifungal [204], photoprotective [205,206,207], antioxidant [208,209], and anti-inflammatory [210,211] effects. In topical formulations, ferulic acid improves stability and bioavailability in the therapy of skin disorders [212]. Moreover, it can be an effective photoprotective cosmetic ingredient [213]. Ferulic acid nanoparticles accelerated wound healing in diabetic patients [214]. In combination with berberine, ferulic acid served as a promoter of cellular clearance through the expression of Sirt1 and AMPK, thereby promoting longevity [215]. As a strong antioxidant, this acid can be used in the phytotherapy of many oxidative stress-related diseases, e.g., Alzheimer’s disease [216,217,218], diabetes [217,219,220,221], atherosclerosis [222,223], hypertension [224], and cancer [225,226,227].

### 3.9. Flavonoids

In the present study, the flavonoids found in the *Salvae herba* of the analysed taxa were dominated by apigenin in *S. officinalis* (265.14 μg·mL^−1^) and *S. officinalis* subs. *lavandulifolia* (221.60 μg·mL^−1^) and by kaempferol in *S. sclarea* (313.64 μg·mL^−1^). The health-enhancing potential of apigenin in various biological models was reflected in its antibacterial [228,229], antiviral [230,231], antifungal [232], antioxidant [159,168,233,234], anti-inflammatory [235,236,237], photoprotective [238,239], healing [240,241,242], anticancer [243,244], anti-apoptotic [245,246,247,248], antiangiogenic [249,250,251], antiproliferative [247,250,252], immunomodulatory [250,253,254], and neuroprotective [188,255,256], activity. Furthermore, it has been documented that apigenin has the following effects: anti-diabetic [257,258], anti-obesity [259,260], cardioprotective [261,262,263], antidepressant [264,265,266], anti-insomnia [267], and anti-dementia [268] activity and supports the therapy of Alzheimer’s disease [269,270]. This indicates that apigenin has a promising future. Supplementation with this compound may have pro-health effects in many diseases.

Kaempferol was determined in the present study to range from 175.32 (*S. officinalis*) to 313.64 μg·mL^−1^ (*S. sclarea*). The value of this parameter in these species reported by Dziadek et al. [67] was 2.21 and 2.71 mg∙100 g^−1^ DW, respectively. The kaempferol content in various extracts of *S. officinalis* leaves was in the range of 1.90–3.4 mg·L^−1^ [271]. As reported by Kharazian [272], the total percentage share in the pool of identified polyphenolic compounds in *S. sclarea* leaves was 7.60%. Kaempferol, i.e., a naturally occurring aglycone dietary flavonoid, is present in various plants [273]. Alrumaihi et al. [274] presented the multidirectional biological effects of kaempferol in the treatment of pathogenic conditions, i.e., modulation of inflammation and other biological activities. Kaempferol and epicatechin inhibited the growth of *Helicobacter pylori* in a concentration-dependent manner [275]. As reported by Gao et al. [276], kaempferol in combination with azithromycin alleviated *Staphylococcus aureus*-induced osteomyelitis through inhibition of ERK1/2 and SAPK phosphorylation. Shao et al. [277] showed that kaempferol had antifungal activity against *Candida albicans* through inhibition of the expression of *CDR1*, *CDR2*, and *MDR1*. Kaempferol has been documented to act against *Giardia duodenalis*-induced gardiasis through its proapoptotic effect on *G. duodenalis* trophozoites, causing DNA synthesis disorders without oxidative stress or damage to chromatin structure and cytoskeletal structures [278]. Kaempferol interacts with pleiotropic proteins in humans, acts as one of the modulators of the immune system, and participates in the prevention of hepatocellular carcinoma as an antioxidant [273]. Kaempferol has been confirmed to be effective in inhibiting ovarian, breast, and lung cancer, which is related to the mechanisms of anti-inflammatory and antioxidant action. It induces apoptosis in cancer cells and inhibits the growth and relocation of cancer cells [279]. Kaempferol exhibits anti-inflammatory properties and improved inflammatory markers. The problem of its poor bioavailability has been solved with the use of nanotechnology [280]. Kaempferol exerted anti-inflammatory effects in *Helicobacter pylori* infection associated with gastric carcinogenesis. It inhibited the translocation of CagA and VacA proteins and reduced the expression of pro-inflammatory cytokines (TNF-α, IL-1β, and IL-8) [281]. Additionally, it reduced CD3 T cell infiltration and the expression of genes of the key pro-inflammatory cytokines, including interleukin (IL)-6, IL-17A, and tumour necrosis factor (TNF)-α, in psoriatic skin lesions. Its potential as an active chemical compound in psoriasis therapy requires further investigation [282].

### 3.10. Quinic Acid

In the present study, quinic acid was identified in the three species at a concentration in the range from 253.84 μg·mL^−1^ (*S. officinalis* subsp. *lavandulifolia*) to 346.69 μg·mL^−1^ (*S. sclarea*). As indicated by literature reports, the content of this acid in sage leaves constituted 1.19% of the total amount of compounds [283]. Quinic acid is present in many edible fruits and plants. Under the influence of the gastrointestinal microflora, it is converted into essential tryptophan and nicotinamide. It increases their biosynthesis, thus strengthening DNA and reducing NF-kB, and induces antioxidant metabolism in the human organism [284]. Quinic acid was reported to have antibacterial effects on Gram-negative bacteria: *Escherichia coli* and *Klebsiella pneumonia* and Gram-negative bacteria: *Staphylococcus aureus* and *S. pyogenes* [285]. This property is associated with the regulation of ribosome function and amino acyl-tRNA synthesis, modification of fatty acids and glycerophospholipids, and disruption of the oxidative phosphorylation pathway resulting in membrane fluidity [286]. Quinic acid with high water solubility exhibited the highest antioxidant activity in the DMPD method, compared to other methods [285]. It increased the activity of the antioxidant enzyme superoxide dismutase (SOD) and the level of oxidative stress markers and glutathione (GSH). It also inhibited the level of malondialdehyde (MDA) in the SH-SY5Y (neuroblastoma) cell line and increased the levels of catalase (CAT) and glutathione peroxidase (GPx). Quinic acid reduced the expression of inflammatory interleukin-1β (IL-1β) and tumour necrosis factor α (TNF-α) in SH-SY5Y cells. It protected nerve cells against damage through inhibition of oxidative stress and inflammation, thereby preventing oxidative stress-related diseases [287]. It reduced the symptoms of neuroinflammation through regulation of the activation of the pro-inflammatory mediator and the phosphorylation of extracellular signal-regulated kinase (ERK) in astrocytes [288]. It counteracted brain oxidative stress and neuroinflammation induced by a high-fat diet by regulation of the inflammatory DR3/IKK/NF-κB signalling pathway via tryptophan metabolites [289]. Quinic acid had an antidiabetic effect, as it stimulated insulin secretion through mobilisation of Ca^2+^ from intracellular reserves and increasing the NAD(P)H/NAD(P)+ ratio [286]. Its anticancer effect consisted in increasing the apoptosis of oral cancer cells (SCC-4) by inhibition of the expression of antiapoptotic genes and cyclin D1 as well as cell proliferation [290]. The acid inhibited the activator protein 1 (AP-1) and PKC signalling pathways and downregulated the expression of matrix metallopeptidase 9 (MMP-9) [286]. Quinic acid derivatives can be used in the fight against dengue virus infection [291].

## 4. Future Research

Future studies are recommended to determine the concentration of active chemical ingredients in *Salvia* herb, taking into account their toxicity and drug–drug interactions, and elucidate the molecular mechanisms of their promising therapeutic action. There is a strong demand for future clinical studies confirming the potential antioxidant, anti-inflammatory, and many other properties of sage and its bioactive components, which should be considered in future therapeutic research in humans. Moreover, in the search for such new potential bioactive compounds of natural origin that can effectively and safely support conventional therapies of acute and chronic diseases, further studies focused on specific natural active substances should be based in greater detail on taxonomy and phylogenetic analysis, taking into account related species or taxa from the same clades, which may also contain the compounds analysed in the present study.

## 5. Material and Methods

### 5.1. Plant Material

The study was conducted using *Salviae herba* from *S. officinalis* L., *S. officinalis* subsp. *lavandulifolia* (Vahl) Gams, and *S. sclarea* L. The plants were cultivated on an experimental plot (48.7994442 N, 16.7985236 E) of Mendel University in Brno, Faculty of Horticulture, Department of Vegetable Growing and Floriculture, in Lednice, a town located in the South Moravian region of the Czech Republic. The plant material was collected manually from each taxon in the initial flowering phase in the second decade of June 2024. The plants were randomly selected from the entire surface of the plot. The collected *Salviae herba* raw material was dried in standardised conditions (40 °C). The dried material was ground in a laboratory mill IKA A 11 Basic (IKA Werke GmbH & Co. KG, Staufen, Germany), and samples were taken to determine the content of selected chemical compounds [292,293]. The solvents and chemicals used for the analyses were of liquid chromatography, gas chromatography or analytical grade.

### 5.2. Moisture

The moisture content in the *Salviae herba* samples collected from *S. officinalis*, *S. officinalis* subsp. *lavandulifolia*, and *S. sclarea* was determined with the gravimetric method using a Radwag AS 310.X2 PLUS analytical balance (Radwag, Radom, Poland), with a readability of d = 0.0001 g and a maximum weighing range of 310 g. Samples weighing 2 g were placed in glass laboratory vessels with a glass lid and dried in a Memmert UFE 500 type laboratory dryer (Memmert, Lilienthal, Germany) to constant weight at 105 °C. The dry mass of a given sample was calculated in % by dividing the weight of the residue after drying by the weight of the sample × 100 [294].

$$\mathrm{Dry}\;\mathrm{weight}\;(\%)=\frac{weight\;of\;sample\;dried\;at\;105\;{}^{\circ}C\;\left(g\right)\times 100}{sample\;weight\;before\;drying\;(g)}$$

### 5.3. Determination of Ash Content

The percentage weight of ash in the *Salviae herba* samples collected from *S. sclarea*, *S. officinalis*, and *S. officinalis* subsp. *lavandulifolia* was calculated using the gravimetric method. Samples weighing 2 g were placed in quartz crucibles, dried, carbonised, and incinerated at 550 °C in a muffle furnace FCF 12SHM (Czylok, Jastrzębie Zdrój, Poland). After cooling, the weight of the residue was determined. The ash weight (%) was calculated by dividing the weight of the residue by the weight of the sample × 100 [295].

$$\%\;\mathrm{ash}\;\mathrm{weight}=\frac{sample\;weight\;after\;icineration\;at\;550\;{}^{\circ}C\;\left(g\right)\times 100}{sample\;weight\;before\;icineration\;(g)}$$

### 5.4. Determination of Total Nitrogen and Protein Content

The total nitrogen content in the *Salviae herba* of *S. officinalis* L., *S. officinalis* subsp. *lavandulifolia*, and *S. sclarea* was determined using the Kjeldahl method [296]. This method consists in conversion of organic nitrogen compounds into ammonium sulphate using concentrated sulphuric acid in the presence of a copper catalyst, alkalisation of the solution, distillation, and titration of the ammonia bound by boric acid with hydrochloric acid. Mineralisation of the plant material was performed using a Tecator Digestor Auto 20 mineraliser (FOSS, Hilleroed, Denmark). The distillation and titration processes were carried out using an automatic KD310-A-1015 KjelROC Analyser (OPSIS Liquid LINE, Furulund, Sweden), sodium hydroxide at a concentration of 40%, a receiving acid solution for automatic titration—boric acid 1% with a solution of bromocresol green and methyl red as an indicator.

The percentage nitrogen content was calculated automatically according to the formula:

$$\%\;\mathrm{nitrogen}\;=\;\frac{\left(T-B\right)\times N\times 14,007\times 100}{sample\;weight\;(mg)}$$

where T and B are volumes (mL) of acid used to titrate the sample and the blank, and N (Eq/L) is the normality of boric acid.

The total protein content was calculated by multiplying the nitrogen content by the protein coefficient of 6.25 [297].

### 5.5. Qualitative and Quantitative Analysis of Amino Acids

The composition of amino acids in the *Salviae herba* raw material collected from *S. sclarea*, *S. officinalis*, and *S. officinalis* subsp. *lavandulifolia* was determined according to the method developed by Davies and Thomas [298]. Plant material samples (n = 3) were placed in an INGOS hydrolyser thimble (Prague, Czech Republic) and flooded with 6M HCl. After closing the valve, the solution was saturated with nitrogen and hydrolysis was carried out at 110 °C for 20 h. The content of the thimble after hydrolysis was cooled and filtered through a G-4 funnel. The hydrolysate was evaporated on a RVO 400 SD vacuum evaporator at 50 °C, washed with 1 mL of distilled water, and evaporated again. The dry residue from the vacuum flask was dissolved in 5 mL of citrate buffer pH 2.2. The sample was dosed onto a 35 cm long column with a diameter of 5 mm filled with ion exchange resin. The separation of amino acids was carried out using an AAA 400 amino acid analyser at a temperature of T_1_ = 60 °C and T_2_ = 63 °C. Individual amino acids were derivatised into coloured amino acid-ninhydrin complexes. They were identified using a photometric detector at a wavelength of 570 nm or 440 nm (for proline). The measurement was recorded as a chromatogram using the CHROMuLAN software v0.79 (INGOS, Prague, Czech Republic).

### 5.6. Determination of Fat Content

The total fat content was determined with the Soxtec™ approach based on the classic Soxhlet method, i.e., an innovative liquid-solid solvent extraction technique using the Randall immersion method [299]. The samples were ground separately in a stainless-steel grinder, dried in a Memmert UFE 500 laboratory dryer (Memmert, Lilienthal, Germany) at 103 C, weighed into extraction thimbles, and placed in the Soxtec Avanti^®^ extraction unit (Tecator, Buchi, Switzerland). The extraction vessels were weighed, filled with n-hexane, and the soluble material was extracted in a two-step process, followed by a solvent recovery phase. Next, the extraction vessels with fat were dried in a Memmert UFE 500 type laboratory dryer (Memmert, Lilienthal, Germany) and weighed. The fat content (%) was calculated based on the known fat weight and sample weight using the formula:

$$\%\;\mathrm{fat}\;=\;\frac{W3-W2\times 100\%}{W1}$$

W1—dry sample weight (g); W2—extraction vessel weight (g); W3—extraction vessel weight with fat (g).

### 5.7. Qualitative and Quantitative Analysis of Fatty Acids

The qualitative and quantitative analysis of fatty acids in the *Salviae herba* obtained from *S. sclarea*, *S. officinalis*, and *S. officinalis* subsp. *lavandulifolia* consisted of the three following steps: saponification, esterification of fatty acids, separation and drying, and chromatographic separation. This analysis was performed in accordance with ISO 12966-1:2014 [300]. In the first step, an approximately 100-mg fat sample was collected using an automatic pipette. A methanolic solution of potassium hydroxide was used in the saponification process, and a methanolic solution of boron trifluoride was added in the esterification process. The separation process was carried out with the use of hexane and a saturated sodium chloride solution. The hexane layer was collected in a glass vial and dried over anhydrous sodium sulphate. The chromatographic analysis was carried out using a Varian 450-GC (Temecula, CA, USA) gas chromatograph equipped with a 1177 Split/Splitless injector at 250 °C with a Select™ Biodiesel for FAME capillary column (30 m; 0.32 mm; 0.25 μm). The stationary phase included Select Biodiesel for FAME Fused Silica, a column oven with an initial temperature of 100 °C and a final temperature of 240 °C, and a FID detector (temperature 270 °C). The helium carrier gas flow rate was 1.5 mL/min. The results were calculated using the Galaxie™ Chromatography Data System v1.9.3 software controlling the CP-8400 autosampler.

### 5.8. Determination of Ascorbic Acid Content

Crushed samples weighing 2.5 g were transferred to 50-mL volumetric flasks. After addition of approximately 40 mL of metaphosphoric acid at a concentration of 20 g·L^−1^, the content was shaken for extraction and then made up to a volume of 50 mL. Immediately after the extraction, 20 mL of the extract was transferred into a 100 mL beaker, 10 mL of a cysteine solution was added, and the mixture was stirred on a magnetic stirrer. By adding a solution of trisodium phosphate at a concentration of 200 g·L^−1^, the pH value was adjusted to pH 7.00–7.20 using a Mettler Toledo FiveEasy PLUS FP20 pH meter (Mettler-Toledo, Warsaw, Poland). After mixing with a solution of concentrated metaphosphoric acid at a concentration of 200 g·L^−1^, the pH was lowered to 2.50–2.80. The content of the beaker was added quantitatively to 50-mL volumetric flasks and made up with deionised water to the mark. After filtering through a fluted filter and a syringe filter, it was dosed onto the column. The determination was carried out on a Shimadzu chromatograph with a DAD detector (Shimadzu, Kyoto, Japan). The mobile phase was a 0.1 M phosphoric acid solution with pH 2.80. A Gemini C18 column 150 × 4.4 mm with a grain diameter of 5 μm was used at a temperature of 20 °C. Vitamin C was determined using the external standard method at a wavelength of 245 mn PN-EN 14130:2004 [301].

### 5.9. Polyphenolic Compounds

The content of polyphenolic acids, *ortho*-dihydroxyphenols, and flavonoids was determined in the *Salviae herba*.

#### 5.9.1. Content of Total Ortho-Dihydroxyphenols

The determination of the content of total ortho-dihydroxyphenols involved the extraction of active substances contained in the analysed material and measurement of the absorbance of the solutions at a wavelength of λ = 725 nm [302]. The extraction process consisted in dissolving a 0.5-g sample in anhydrous methanol (50 mL). Next, 1 mL of the extract was placed in a 25-mL flask, and 2 mL of methanol, 10 mL of deionised water, and 2 mL of Folin–Ciocalteu were added. After three minutes, 1 mL of 10% Na_2_CO_3_ was added and, after mixing, the sample was allowed to stand for 30 min. After this time, the flask was filled with deionised water to the mark and measurements were performed spectrophotometrically using a Shimadzu 1800 device (Shimadzu Corp., Kyoto, Japan). A series of standard solutions for the calibration curve were prepared from a stock solution of caffeic acid (concentration: 1.00 mg·ml^−1^).

#### 5.9.2. Total Flavonoid Content

The total content of flavonoids in the *Salviae herba* collected from *S. sclarea*, *S. officinalis*, and *S. officinalis* subsp. *lavandulifolia* was determined in stock, test, and reference solutions [303]. The stock solution was prepared in the first step, i.e., an aliquoted sample of the raw material was transferred into a flask and 20 mL of acetone, 2 mL of hydrochloric acid, and 1 mL of methenamine solution were added. The mixture was kept in a boiling water bath under a reflux condenser for 30 min. The hydrolysate was filtered into a volumetric flask (100 mL) and supplemented with acetone. After placing 20 mL of the solution in the separatory funnel, 20 mL of water was added and the content was extracted with 15 mL of ethyl acetate and three times with 10 mL. Combined organic layers were washed twice with water (40 mL each), filtered into a volumetric flask (50 mL), and supplemented with ethyl acetate.

Next, the test solution was prepared as follows: 2 mL of an aluminium chloride solution (20 g·l^−1^) was added to 10 mL of the stock solution and supplemented with a mixture (1:19) of acetic acid with methanol to 25 mL.

To prepare the reference solution, 10 mL of the stock solution supplemented with a mixture (1:19) of acetic acid with methanol to 25.0 mL was used. After 45 min, the absorption of the solutions was measured at 425 nm using the reference solution for comparison. The percentage of flavonoids was calculated as quercetin equivalent according to the formula:

$$\%\;\mathrm{flavonoids}\;=\;\frac{A\times k}{m}$$

where A is the absorption of the test solution, k is the conversion factor for quercetin (k = 0.875), and m is the raw material aliquot (g).

### 5.10. Qualitative and Quantitative Analysis of Phenolic Acids and Flavonoids

#### 5.10.1. Sample Preparation Procedure

Samples (300 mg) of finely ground dried plant material with 3 mL of methanol in a glass vial were sonicated in an ultrasonic bath (Polsonic Instrument SONIC-2, 250 W (Polsonic, Poland)) at 40 °C for 60 min. After centrifugation at 2600 rpm for 10 min using a MPW 341 centrifuge (MPW Med. Instruments, Warsaw, Poland), the supernatant was filtered and subjected to LC/MS analysis.

#### 5.10.2. HPLC Measurements

The chromatographic measurements were performed on a LC/MS system consisting of a UHPLC chromatograph (UltiMate 3000, Dionex, Sunnyvale, CA, USA), a linear trap quadrupole-Orbitrap mass spectrometer (Q-Exactive from Thermo Fisher Scientific, San Jose, CA, USA), and an ESI source. A Kinetex column (4.6 × 100 mm, 2.6 μm) (Phenomenex, Torrance, CA, USA) was used for chromatographic separation performed using gradient elution. 25 mM formic acid in water was mobile phase A, and 25 mM formic acid in acetonitrile was mobile phase B. The gradient program started at 5% B increasing to 95% for 45 min and the next isocratic elution followed (95% B) for 15 min. The total run time was 60 min at the mobile phase flow rate of 0.4 mL/min. In the course of each run, PDA spectra in the range of 190–600 nm and MS spectra in the range of 100–1000 *m*/*z* were collected continuously.

The ESI was operated in negative polarity modes in the following specific conditions: spray voltage—3.5 kV; sheath gas—40 arbitrary units; auxiliary gas—10 arbitrary units; sweep gas—10 arbitrary units; and capillary temperature—320 °C. Nitrogen (>99.98%) was used as sheath, auxiliary, and sweep gas. The scan cycle used a full-scan event at the resolution of 70,000.

### 5.11. Statistical Analysis

The significance of differences in the levels of the compounds in the chemical profiles in three *Salviae herba* taxa (n = 3), i.e., ash, protein, fat, ascorbic acid, flavonoids, and ortho-dihydroxyphenols, and the qualitative and quantitative analysis of protein amino acids, fatty acids, phenolic acids, flavonoids, malic acid, and quinic acid were analysed statistically using the integrated statistical and analytical software package SAS 9.2 and Statistica 6.0. The analysis of variance (ANOVA) and Tukey’s comparison tests were performed at the significance level of α = 0.05.

## 6. Conclusions

Among the three analysed *Salvia* herbs, *S. sclarea* was characterised by the lowest content of total protein (8.57%) and fat (3.84%), while the highest levels of these compounds were found in *S. officinalis* subsp. *lavandulifolia* (16.14% and 7.80%, respectively). In terms of the content of exogenous protein amino acids, the species were ranked as follows (mg·g^−1^ DW): *S. sclarea* (26.1) *< S. officinalis* subsp. *lavandulifolia* (44.0) < *S. officinalis* (53.3). Leucine, phenylalanine, and valine were the dominant exogenous amino acids in the *Salviae herba* and they were far less abundant in *S. sclarea* than in the other two taxa. *S. officinalis* subsp. *lavandulifolia* was the richest source of saturated (SFA) and unsaturated (MUFA, PUFA) fatty acids. SFAs were dominated by palmitic, stearic, myristic, arachidic, and lignoceric acids. In the group of MUFAs, the sum of oleic and elaidic acids was the most abundant in the herb of the three examined taxa. The range of the content of essential PUFAs was as follows (g·100 g^−1^ DW): omega-3 0.41–1.84, omega-6 0.78–0.24, and omega-9 0.37–0.21. *S. officinalis* subsp. *lavandulifolia* was the richest source of omega-3 and omega-9 acids, while omega-6 acids were the most abundant in *S. officinalis*. *S. sclarea* was shown to be the poorest source of these fatty acids. The omega-3 family was dominated by α-linolenic acid together with eicosapentaenoic acid and docosahexaenoic acid. In the omega-6 class, the sum of linoleic and linoelaidic acids was the most abundant together with *Z*-11,14-eicosadienoic acid, 13,16-docosadienoic acid, and dihomo-γ-linolenic acid, while the omega-9 group was dominated by the sum of oleic and elaidic acids as well as eruic acid. There were differences among the three examined taxa in the qualitative composition of SFAs in the case of tridecanoic acid, heneicosylic acid, and tricosanoic acid. The first acid was detected only in *S. officinalis* subsp. *lavandulifoila*, the second in *S. sclarea*, and the third in *S. officinalis*. In the MUFA family, the presence of 10-pentadecenoic acid and nervonic acid was shown only in the herb of *S. sclarea*. In the PUFA group, γ-linolenic acid and arachidonic acid were present only in *S. sclarea*, while *Z*-11,14,17-eicosatrienoic acid was detected only in *S. officinalis* subsp. *lavandulifolia*. The highest content of rosmarinic acid, i.e., the most abundant of all the phenolic compounds, was found in *S. sclarea*, while its level in the other two taxa was comparable. Considering the content of the most common hydroxy derivatives of cinnamic acid, the lowest level of *p*-coumaric acid was recorded in *S. officinalis*, caffeic acid in *S. sclarea*, and ferulic acid in *S. officinalis* subsp. *lavandulifolia*, while their highest levels were determined in *S. sclarea*, *S. officinalis* subsp. *lavandulifolia*, and *S. officinalis*, respectively. *S. officinalis* was characterised by the lowest content of chlorogenic acid, whereas moderate and the highest levels of this acid were determined in *S. sclarea* and *S. officinalis* subsp. *lavandulifolia*, respectively. The levels of salicylic acid were similar in *S. officinalis* and *S. sclarea* and markedly exceeded those found in *S. officinalis* subsp. *lavandulifolia*. The content of vanillin and coumarin in *S. officinalis* and *S. sclarea* were similar and lower than in *S. officinalis* subsp. *lavandulifolia*. Considering benzoic acid derivatives, the lowest amount of gallic acid was found in *S. officinalis*, and its highest content was determined in *S. officinalis* subsp. *lavandulifolia*, while the opposite tendency was found for the content of protocatechuic acid. Among the three taxa, the lowest content of non-phenolic organic acids, i.e., malic and quinic acids, was recorded in *S. officinalis* subsp. *lavandulifolia*, while their highest level was detected in *S. officinalis*. *S. officinalis* subsp. *lavandulifolia* (11.52 mg·100 g^−1^ DW) was a considerably richer source of vitamin C than *S. sclarea* (4.87 mg·100 g^−1^ DW) but poorer than *S. officinalis* (14.43 mg·100 g^−1^ DW). The total level of *o*-dihydroxyphenols in the *S. officinalis* subsp. *lavandulifolia* herb (2179 mg CAE·100 g^−1^ DW) was notably higher than in *S. officinalis* (2140 mg·100 g^−1^ DW) but lower than in *S. sclarea* (2222 mg CAE·100 g^−1^ DW). The highest level of flavonoids was detected in *S. officinalis* subsp. *lavandulifolia* (610 mg RU·100 g^−1^ DW), but the lowest content of these metabolites, which can be used as components of potential phytotherapy products, was found in *S. sclarea* (347 mg RU·100 g^−1^ DW). Considering the flavonoid subclasses, within the flavone group, *S. sclarea* contained far less apigenin and more luteolin than *S. officinalis* subsp. *lavandulifoila* and *S. officinalis*, but the chrysin content was comparable in all these taxa. The contents of flavanones (hesperetin, hesperidin, and naringenin) determined in *S. sclarea* notably exceeded their level found in the other two taxa. Within the subclass of flavonols, *S. sclarea* was characterised by substantially higher content of fisetin, hyperoside, kaempferol, quercetin, and rhamnetin and substantially lower levels of myricetin and rutin than *S. officinalis* subsp. *lavandulifoila* and *S. officinalis*. The rich chemical profile in *Salviae herba*, including amino acids, contributes not only to protein structural functions but also to nitrogen metabolism, enzyme synthesis, and immune stimulation, thereby supporting regenerative and metabolic processes in the human organism upon ingestion. The presence of polyunsaturated fatty acids has an impact on the composition of membrane phospholipids as well as the regulation of lipid metabolism and the activity of pro-inflammatory cytokines, which is associated with a reduction in inflammatory responses. Furthermore, phenolic acids, flavonoids, and other identified phenolic compounds exhibit potent antioxidant and anti-inflammatory activities through inhibition of reactive oxygen species and modulation of pro-inflammatory pathways via suppression of cytokine expression. The chemical profile of *Salviae herba* supports its use as a phytotherapeutic raw material with potential applications in management of chronic inflammation, metabolic disorders, and oxidative stress, owing to the antioxidant, anti-inflammatory, and metabolism-modulating properties of the bioactive compounds contained in the analysed *Salvia* taxa.

## Figures and Tables

**Table 1:** Nomenclature, abbreviations, symbols, and formulas of amino acids contained in *Salviae herba*.

Trivial Name	IUPAC Name: Systematic:	Abbreviation	Symbol	Molecular Formula	Linear Chemical Formula	Structural Chemical Formula
Arginine	(*S*)-2-Amino-5-guanidinopentanoic acid	Arg	R	C_6_H_14_N_4_O_2_	HN=C(NH_2_)-NH-(CH_2_)_3_-CH(NH_2_)-COOH	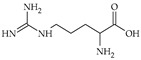
Phenylalanine	(2*S*)-2-amino-3-phenylpropanoic acid	Phe	F	C_9_H_11_NO_2_	C_6_H_5_CH_2_CH(NH_2_)CO_2_H	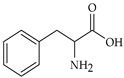
Histidine	(2*S*)-2-amino-3-(1*H*-imidazol-5-yl)propanoic acid	His	H	C_6_H_9_N_3_O_2_	NH-CH=N-CH=C-CH_2_-CH(NH_2_)-COOH	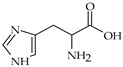
Isoleucine	(2*S*,3*S*)-2-amino-3-methylpentanoic acid	Ile	I	C_6_H_13_NO_2_	C_2_H_5_CH(CH_3_)CH(NH_2_)CO_2_H	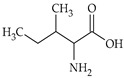
Leucine	(2*S*)-2-amino-4-methylpentanoic acid	Leu	L	C_6_H_13_NO_2_	(CH_3_)_2_CHCH_2_CH(^15^NH_2_)CO_2_H	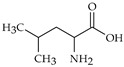
Lysine	(2*S*)-2,6-diaminohexanoic acid	Lys	L	C_6_H_14_N_2_O_2_	H_2_N-(CH_2_)_4_-CH(NH_2_)-COOH	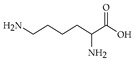
Threonine	(2*S*,3*R*)-2-amino-3-hydroxybutanoic acid	Thr	T	C_4_H_9_NO_3_	CH_3_CH(OH)CH(^15^NH_2_)CO_2_H	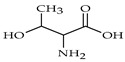
Valine	(2*S*)-2-amino-3-methylbutanoic acid	Val	V	C_5_H_11_NO_2_	(CH_3_)_2_CHCH(NH_2_)CO_2_H	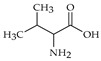
Alanine	2-aminopropanoic acid	Ala	A	C_3_H_7_NO_2_	CH_3_CH(NH_2_)COOH	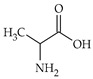
Glycine	2-Aminopropanoic acid	Gly	G	C_2_H_5_NO_2_	NH_2_CH_2_COOH	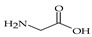
Aspartic acid	2-Aminobutanedioic acid	Asp	D	C_4_H_7_NO_4_	HO_2_CCH_2_CH(NH_2_)CO_2_H	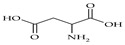
Glutamic acid	2-Aminopentanedioic acid	Glu	E	C_5_H_9_NO_4_	HO_2_CCH_2_CH_2_CH(NH_2_)CO_2_H	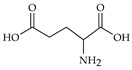
Proline	Pyrrolidine-2-carboxylic acid	Pro	P	C_5_H_9_NO_2_	HO_2_CCH(NH[CH_2_)_3_	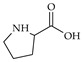
Serine	(2*S*)-2-amino-3-hydroxypropanoic acid	Ser	S	C_3_H_7_NO_3_	HOCH_2_CH(_15_NH_2_)CO_2_H	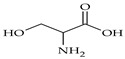
Tyrosine	(2*S*)-2-amino-3-(4-hydroxyphenyl)propanoic acid	Tyr	Y	C_9_H_11_NO_3_	4-(OH)C_6_H_4_CH_2_CH(_15_NH_2_)CO_2_H	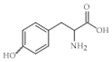

**Table 2:** Nomenclature, abbreviations, and formulas of saturated fatty acids (SFAs) contained in the herb of the analysed *Salvia* species.

Trivial Name	IUPAC Name: Systematic:	Abbreviation	Molecular Formula	Linear Chemical Formula	Structural Chemical Formula
Caproic acid	Hexanoic acid	C6:0	C_6_H_12_O_2_	CH_3_(CH_2_)_4_COOH	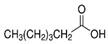
Caprylic acid	Octanoic acid	C8:0	C_8_H_16_O_2_	CH_3_(CH_2_)_6_COOH	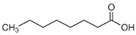
Capric acid	Decanoic acid	C10:0	C_10_H_20_O	CH_3_(CH_2_)_8_COOH	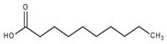
Hendecanoic acid	Undecanoic acid	C11:0	C_11_H_22_O_2_	CH_3_(CH_2_)_9_COOH	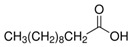
Lauric acid	Dodecanoic acid	C12:0	C_10_H_20_O_2_	CH_3_(CH_2_)_10_COOH	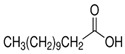
Tridecylic acid	Tridecanoid acid	C13:0	C_13_H_26_O	CH_3_(CH_2_)_11_CO_2_H	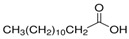
Myristic acid	Tetradecanoic acid	C14:0	C_14_H_28_O_2_	CH_3_(CH_2_)_12_COOH	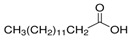
Pentadecylic acid	Pentadecanoic acid	C15:0	C_15_H_30_O_2_	CH_3_(CH_2_)_13_COOH	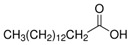
Palmitic acid	Hexadecanoic acid	C16:0	C_16_H_32_O_2_	CH_3_(CH_2_)_14_COOH	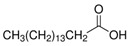
Margaric acid	Heptadecanoic acid	C17:0	C_17_H_34_O_2_	CD_3_(CD_2_)_14_CD_2_COOH	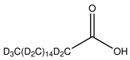
Stearic acid	Octadecanoic acid	C18:0	C_18_H_36_O_2_	CH_3_(CH_2_)16COOH	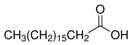
Arachidic acid	Eicosanoic acid	C20:0	C_18_H_36_O_2_	CH_3_(CH_2_)_18_COOH	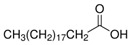
Heneicosylic acid	Heneicosanoic acid	C21:0	C_21_H_42_O_2_	CH_3_(CH_2_)_19_COOH	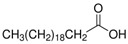
Behenic acid	Docosanoic acid	C22:0	C_22_H_44_O_2_	CH_3_(CH_2_)_20_COOH	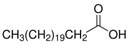
Tricosylic acid	Tricosanoic acid	C23:0	C_23_H_46_O_2_	CH_3_(CH_2_)_21_COOH	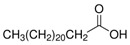
Lignoceric acid	Tetracosanoic acid	C24:0	C_24_H_48_O_2_	CH_3_(CH_2_)_22_COOH	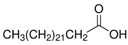

Explanation: nd—not determined.

**Table 3:** Nomenclature, abbreviations, and formulas of monounsaturated fatty acids (MUFAs) contained in the herb of the analysed *Salvia* species.

Trivial Name	IUPAC Name: Systematic:	Abbreviation	Omega	Molecular Formula	Linear Chemical Formula	Structural Chemical Formula
Myristoleic acid	(9*Z*)-tetradec-9-enoic acid	C14:1n5	Omega-5	C_14_H_26_O_2_	CH_3_(CH_2_)_3_CH=CH(CH_2_)_7_COOH	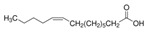
*cis*-10-pentadecenoic acid	(10*Z*)-pentadec-10-enoic acid	C15:1n5	Omega-5	C_15_H_28_O_2_	CH_3_(CH_2_)_4_CH=CH (CH2)_8_COOH	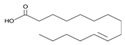
Palmitoleic acid	(9*Z*)-hexadec-9-enoic acid	C16:1n7	Omega-7	C_16_H_30_O_2_	CH_3_(CH_2_)_5_CH=CH(CH_2_)_7_COOH	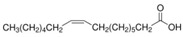
*cis*-10-heptadecenoic acid	(10*Z*)-heptadec-10-enoic acid	C17:1n7	Omega-7	C_17_H_32_O_2_	CH_3_(CH_2_)_15_COOH	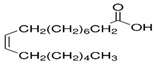
Oleic acid	(9*Z*)-octadec-9-enoic acid	C18:1n9c	Omega-9	C_18_H_34_O_2_	CH_3_(CH_2_)_7_CH=CH(CH_2_)_7_COOH	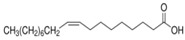
Elaidic acid	(9*E*)-octadec-9-enoic acid	C18:1n9t	Omega-9	C_18_H_34_O_2_	CH_3_(CH_2_)_7_CH=CH(CH_2_)_7_COOH	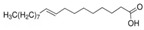
*cis*-5-eicosenoic acid	(5*Z*)-eicos-5-enoic acid	C20:1n15	Omega-9	C_20_H_38_O_2_	CH_3_(CH_2_)_7_CH=CH(CH_2_)_9_COOH	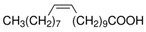
Erucic acid	(13*Z*)-docos-13-enoic acid	C22:1n9	Omega-9	C_22_H_42_O_2_	CH_3_(CH_2_)_7_CH=CH(CH_2_)_11_COOH	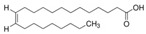
Nervonic acid	(15*Z*)-tetracos-15-enoic acid	C24:1n9	omega-9	C_24_H_46_O_2_	CH_3_(CH_2_)_7_CH=CH(CH_2_)_13_COOH	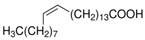

**Table 4:** Nomenclature, abbreviations, and formulas of polyunsaturated fatty acids (PUFAs) contained in the herb of the analysed *Salvia* species.

Trivial Name	IUPAC Name: Systematic:	Abbreviation	Omega	Molecular Formula	Linear Chemical Formula	Structural Chemical Formula
Linoleic acid	(9*Z*,12*Z*)-octadeca-9,12-dienoic acid	C18:2n6c	Omega 6	C_18_H_32_O_2_	CH_3_(CH_2_)_4_CH=CHCH_2_CH=CH(CH_2_)_7_COOH	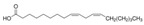
Linoelaidic acid	(9*E*,12*E*)-octadeca-9,12-dienoic acid	C18:2n6t	Omega-6	C_18_H_32_O_2_	CH_3_(CH_2_)_4_CH=CHCH_2_CH=CH(CH_2_)_7_COOH	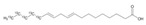
(all-*cis*)-11,14-eicosadienoic acid	(11*Z*,14*Z*)-eicosa-11,14-dienoic acid	C20:2n6	Omega-6	C_20_H_36_O_2_	CH_3_(CH_2_)_4_(CH=CHCH_2_)_2_(CH_2_)_8_COOH	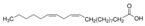
(all-*cis*)-13,16-docosadienoic acid	(13*Z*,16*Z*)-docosa-13,16-dienoic acid	C22:2n6	Omega-6	C_22_H_40_O_2_	CH_3_(CH_2_)_4_(CH=CHCH_2_)_2_(CH_2_)_8_COOH	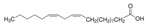
GLA γ-linolenic acid	(6Z,9Z,12Z)-octadeca-6,9,12-trienoic acid	C18:3n6 (gamma)	Omega-6	C_18_H_30_O_2_	CH_3_(CH_2_)_4_CH=CHCH_2_CH=CHCH_2_CH=CH(CH_2_)_4_COOH (all *cis*)	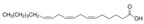
α-linolenic acid	(9*Z*,12*Z*,15*Z*)-octadeca-9,12,15-trienoic acid	C18:3n3 (alpha)	Omega-3	C_18_H_30_O	CH_3_(CH_2_CH=CH)_3_(CH_2_)_7_COOH	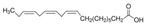
DGLA. Dihomo-γ-linolenic acid	(8*Z*,11*Z*,14*Z*)-eicosa-8,11,14-trienoic acid	20:3n6	Omega-6	C_20_H_34_O_2_	CH_3_(CH_2_)_3_(CH_2_CH=CH)_3_(CH_2_)_6_COOH	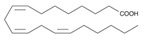
(all-*cis*)-11,14,17-eicosatrienoic acid	(11*Z*,14*Z*,17*Z*)-eicosa-11,14,17-trienoic acid	C20:3n3	Omega-3	C_21_H_36_O_2_	CH_3_(CH_2_)_2_CH=CHCH_2_CH=CHCH_2_CH=CH(CH_2_)_9_COOH	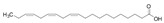
Arachidonic acid	(5*Z*,8*Z*,11*Z*,14*Z*)-eicosa-5,8,11,14-tetraenoic acid	C20:4n6	Omega-6	C_20_H_32_O_2_	CH_3_(CH_2_)_4_(CH=CHCH_2_)_4_CH_2_CH_2_COOH	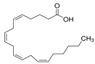
EPA. Timnodonic acid	(5*Z*,8*Z*,11*Z*,14*Z*,17*Z*)-eicosa-5,8,11,14,17-pentaenoic acid	C20:5n3	Omega-3	C_20_H_30_O_2_	CH_3_(CH_2_CH=CH)_5_(CH_2_)_3_COOH	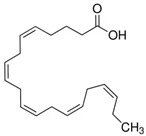
DHA. Cervonic acid	(4*Z*,7*Z*,10*Z*,13*Z*,16*Z*,19*Z*)-docosa-4,7,10,13,16,19-hexaenoic acid	C22:6n3	Omega-3	C_22_H_32_O_2_	CH_3_(CH_2_CHCH)_6_CH_2_CH_2_COOH	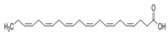

## Data Availability

The data presented in this study are available in this article.
